# The influence of bat ecology on viral diversity and reservoir status

**DOI:** 10.1002/ece3.6315

**Published:** 2020-05-08

**Authors:** Cylita Guy, John M. Ratcliffe, Nicole Mideo

**Affiliations:** ^1^ Department of Ecology and Evolutionary Biology University of Toronto Toronto ON Canada; ^2^ Department of Biology University of Toronto at Mississauga Mississauga ON Canada

**Keywords:** *Chiroptera*, infectious disease forecasting, machine learning, pathogen diversity, viruses, zoonotic disease

## Abstract

Repeated emergence of zoonotic viruses from bat reservoirs into human populations demands predictive approaches to preemptively identify virus‐carrying bat species. Here, we use machine learning to examine drivers of viral diversity in bats, determine whether those drivers depend on viral genome type, and predict undetected viral carriers. Our results indicate that bat species with longer life spans, broad geographic distributions in the eastern hemisphere, and large group sizes carry more viruses overall. Life span was a stronger predictor of deoxyribonucleic acid viral diversity, while group size and family were more important for predicting ribonucleic acid viruses, potentially reflecting broad differences in infection duration. Importantly, our models predict 54 bat species as likely carriers of zoonotic viruses, despite not currently being considered reservoirs. Mapping these predictions as a proportion of local bat diversity, we identify global regions where efforts to reduce disease spillover into humans by identifying viral carriers may be most productive.

## Graphical abstract

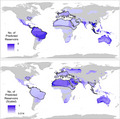

## 1 INTRODUCTION

Bats have been implicated in the transmission of a number of zoonotic diseases (e.g., SARS, Rabies, Nipah, Hendra) that, while often resulting in asymptomatic infections in bats, cause significant mortality in humans and domestic animals (Wang & Anderson, 2019). Given that bats host more viral pathogens per species than other mammalian orders (Luis et al., 2013) and that a greater proportion of their total viral diversity is zoonotic (Olival et al., 2017), there is justified concern over continued disease emergence from this group. There are ~1,400 species of bats (Fenton & Simmons, 2014) making a systematic investigation of viral carriage expensive and, at least in the short term, impractical. However, a viable and immediate alternative is to identify key aspects of bat ecology that underlie observed patterns of viral diversity to predict species that are likely to carry pathogens relevant to human health.

Bats exhibit an extraordinary variety of life‐history strategies (Simmons & Conway, 2003). Several studies have examined how this ecological variation correlates with viral richness (Guy, Thiagavel, Mideo, & Ratcliffe, 2019; Luis et al., 2013; Turmelle & Olival, 2009; Webber, Fletcher, & Willis, 2017). Similar to predictors of parasite diversity in other taxonomic groups (Ezenwa, Price, Altizer, Vitone, & Cook, 2006; Kamiya, O’Dwyer, Nakagawa, & Poulin, 2014; Lindenfors et al., 2007; Nunn, Altizer, Jones, & Sechrest, 2003), traits hypothesized to increase the likelihood of parasite contact and sharing (i.e., larger body sizes, broader geographic distributions, range overlap) are important predictors of increased viral diversity in bats (Guy et al., 2019; Luis et al., 2013; Maganga et al., 2014; Olival et al., 2017). Additionally, bat species with more structured populations (less genetic mixing, possibly facilitating pathogen maintenance in the larger metapopulation) and classified by the International Union for the Conservation of Nature (IUCN) as near‐threatened or vulnerable (which may be more susceptible to infection due to stress) carry a greater number of viruses (Turmelle & Olival, 2009). However, studies have yielded conflicting results about other bat traits. For example, while large group sizes are thought to promote viral transmission, leading to increased pathogen diversity, as observed elsewhere in the literature (reviewed in Patterson & Ruckstuhl, 2013) in bats there is evidence for both positive (Webber et al., 2017) and negative (Gay et al., 2014) associations with viral diversity.

This previous body of research has drawn conclusions from limited subsets of species (Webber et al., 2017, *N* = 51, ~4% of species; Luis et al., 2013, *N* = 66, ~5% of species; Turmell & Olival, 2009, *N* = 33, 3% of species) or restricted geographic areas (Gay et al., 2014, Southeast Asia, *N* = 20, ~2% of species; Maganga et al., 2014, Central and West Africa, *N* = 17, ~1% of species). This is due to limitations of standard phylogenetic comparative methods, which often rely on complete trait information for species included in analyses, and a lack of comprehensive natural history information for many bat species. Machine learning (i.e., algorithms that do not assume an underlying data model but rather learn the relationship between predictors and response; Elith, Leathwick, & Hastie, 2008), with its ability to handle missing data, is a powerful tool for overcoming some of these limitations. Han, Schmidt, Bowden, and Drake (2015) used a machine learning approach to examine drivers of viral diversity in rodents, finding that species with fast‐paced life‐history strategies (i.e., shorter life spans, faster development, smaller bodies) were more likely to be viral carriers. Given the predictive nature of their approach, Han et al. (2015) were able to identify rodent species likely to be carrying zoonotic viruses, despite no viral detections in those species to date. The authors also applied this methodology to bats to predict the distribution of filoviruses, a single viral family that includes Ebola and Marburg (Han et al., 2016). Bat traits such as neonate mass, species’ sympatry, and rates of reproduction were important for predicting the distribution of filoviruses, and 112 bat species were identified as likely, but as yet undetected, filovirus carriers (Han et al., 2016).

Although Han et al. (2016) considered a greater number of bat species (*N* = 1,116) than previous analyses (e.g., Guy et al., 2019; Luis et al., 2013; Turmelle & Olival, 2009; Webber et al., 2017), they examined the distribution of only one (i.e., filoviruses) of the 24 viral families found in bats (Han et al., 2016; Hayman, 2016). Moreover, this body of prior work has not considered how predictors of viral diversity may shift when viral traits are considered. Similar to host traits, the traits of viruses are also likely to govern infection dynamics and transmission between bat species (Geoghegan, Senior, Di Giallonardo, & Holmes, 2016; Luis et al., 2013; Olival et al., 2017). For example, ribonucleic acid (RNA) viruses tend to cause acute infections, that is, they are relatively short‐lived, and often generate long‐lasting immunity (Holmes, 2009; Villarreal, Defilippis, & Gottlieb, 2000). Since large group sizes and synchronized birthing pulses (leading to influxes of susceptible hosts) are likely important for sustaining acute immunizing infections in bats (Calisher, Childs, Field, Holmes, & Schountz, 2006; Hayman, 2015; Plowright et al., 2016), these host traits may be more important for explaining patterns of RNA, compared to deoxyribonucleic acid (DNA), viral diversity.

Here, we extend on previous work to explicitly consider potential interactions between host and viral traits in investigating correlates of viral diversity across the Chiropteran order. We leverage larger species’ trait and viral datasets than previously analyzed and consider traits found to be important drivers of viral diversity in earlier work examining smaller numbers of species (e.g., group size, body size, geographic distribution; Luis et al., 2013; Webber et al., 2017). We also analyze the role of several traits not previously examined (e.g., wing morphology, propensity to form mixed species groups), but hypothesized to influence patterns of viral richness (Calisher et al., 2006; Wang, Walker, & Poon, 2011). In Table A2, we outline our predictions for all traits considered. Using a machine learning approach, we identify the most important bat traits for predicting the richness of viruses they carry and examine if and how the importance of these traits depends on a key viral trait—RNA versus DNA genome. Lastly, we use our models to identify bat species that are likely—though currently undetected—carriers of viruses, highlighting species and geographic regions as key candidates for viral surveillance efforts.

## 2 MATERIALS AND METHODS

### 2.1 Bat species’ trait data

Using primary literature searches and existing databases, we collected ecological trait information for 812 species of bats for which there exists a fully resolved phylogeny (Shi & Rabosky, 2015). Although this is fewer species than analyzed in Han et al. (2016), we included only species for which there are resolved phylogenetic relationships in order to investigate the influence of species’ relationships on inferences (see below). We considered traits previously found to be important predictors of viral diversity in mammals, including: range area (e.g., Lindenfors et al., 2007), latitude of species’ geographic range midpoint (e.g., Lindenfors et al., 2007; Nunn, Altizer, Sechrest, & Cunningham, 2005), torpor expression (e.g., Luis et al., 2013), group size (e.g., Webber et al., 2017), species’ sympatry (e.g., Han et al., 2016; Luis et al., 2013), diet (e.g., Han et al., 2016; Luis et al., 2015), citation count to control for study effort (number of publications for a species’ binomial from Web of Science; e.g., Luis et al., 2013), and forearm size as a proxy for body size (e.g., Kamiya et al., 2014).

We also included several traits not found in previous analyses including number of mixed species roosting associations (potentially facilitating cross‐species transmission), longitude of species’ range midpoint (to separate Old and New World species), and relative wing loading (RWL) and aspect ratio (AR) which characterize wing morphology. RWL and AR also serve as rough proxies for bat ecological niches (Norberg & Rayner, 1987) and capture variation in daily activity patterns. Since body temperature may contribute to pathogen control in bats (O’Shea et al., 2014), activity patterns may influence patterns of viral diversity. For a complete list of the 19 variables considered and pairwise plots of predictors, see Appendix A; Table A1 and Figures A1,A2. Specific hypotheses about predictors' relationships with viral richness are in Table A2.

We considered only those bat species for which we had information on five or more predictor variables (i.e., >25% of the ecological traits considered). This resulted in 747 species (~90% of species in Shi & Rabosky, 2015) included in final models. Supplementary analyses indicate that building models using species for which we have more trait information (e.g., 10 or more ecological traits, 615 species) does not alter conclusions (Appendix B; B1).

### 2.2 Viral data

We collected information on the diversity of viruses hosted by bat species from published records on “DBatVir” (data downloaded April 2018), a continually updated repository of viral sequences (Chen, Liu, Yang, & Jin, 2014). Although previous studies (e.g., Luis et al., 2013; Webber et al., 2017) used the number of viral species hosted by bats as a measure of diversity, we used the number of viral families since viral species classification lags behind surveyed viral diversity (Remita et al., 2017). For each of the viral families in our dataset, we also determined if that family had known zoonotic members (e.g., Coronaviridae, Flaviviridae, Rhabdoviridae).

To investigate whether the importance of bat traits shifts when considering different virus types, for each viral family we recorded genome structure (i.e., RNA, DNA, retrovirus) and Baltimore classification (1971). Using the seven categories of the Baltimore classification partitioned the data too finely to build accurate models (Appendix A, Figure A4), therefore we only considered the influence of viral genome structure (i.e., RNA or DNA, omitting retroviruses) in our analyses. Viral data are summarized in Figures A3–A5.

We used viral data to derive four response variables: total number of viral families, number of viral families with zoonotic members, number of DNA viral families, and number of RNA viral families hosted by bat species. All bat species for which no viral information was present (*n* = 540) were designated as zeros. Supplementary analyses indicate that inclusion of fewer or no zeros does not qualitatively alter conclusions (Appendix B; B.2).

### 2.3 Species’ trait correlates of viral diversity

To examine bat trait correlates of viral diversity, we used boosted regression tree (BRT) models (Elith et al., 2008; Ridgeway, 2017). In BRT models, multiple decision trees are built and combined to improve predictive performance (Elith et al., 2008). BRTs have been used to analyze large species trait datasets (Han et al., 2015, 2016) and are advantageous because they do not assume an underlying data distribution and can handle hidden interactions, different predictor types, and nonrandom patterns of missing data (De'ath & Fabricius, 2000; Elith et al., 2008).

Unlike phylogenetic comparative methods, BRTs do not explicitly control for shared ancestry among species. Considering this, and similar to (Han et al., 2015, 2016), we included family as a predictor in models to explore whether viral carriers are more likely to come from particular bat families. We also used a time‐calibrated phylogeny (Shi & Rabosky, 2015) to group bat species into “phylogenetic clusters” at 41 and 56 million years before present, corresponding to the start of major geological boundaries (the Bartonian age and Eocene Epoch, respectively; Ogg, Ogg, & Gradstien, 2016) and periods of relatively rapid bat speciation (Shi & Rabosky, 2015; Teeling et al., 2005). Using the three phylogenetic groupings (i.e., bat families, 41MYA clusters, and 56MYA clusters), we examined if species’ relationships altered model predictive performance (Appendix B; B.3). Results suggest that while phylogenetic groupings are important for predicting patterns of viral diversity, species’ relationships do not impact model accuracy (i.e., models built on subsets of related species still predict well on subsets of less related species; Appendix B; B.3). Additionally, the importance of bat ecological traits does not depend on which phylogenetic grouping is used (Appendix B; B.3). Models presented in the main text include bat families as a predictor, given that they are more intuitive to interpret.

We performed all analyses in R (*v. 3.4.4*; R Core Development Team, 2014). Using the caret package (*v. 6.0–79*; Kuhn et al., 2018), we split our dataset into training (80% of species) and test (20% of species) sets. For each BRT model, we used the caret package to determine optimal values for the following: learning rate, tree complexity, and number of trees. We used optimized parameter values to build BRT models using 10‐fold cross validation. All BRT models were built with a Poisson loss function using the gbm package (*v. 2.0–8*; Ridgeway, 2017). For each BRT model, we determined the relative importance of each predictor (i.e., its contribution to the final model expressed as a percentage) and computed pseudo *R* ^2^ measures (i.e., a measure of successful predictions to unsuccessful ones) for training and test datasets. Finally, we generated partial dependence plots, which illustrate how individual traits influence viral diversity, holding the effects of other predictors constant (Elith et al., 2008).

We built four BRT models with either total, zoonotic, DNA, or RNA viral family diversity as the response and all ecological traits as predictors. Given variability between BRT model runs (Appendix B), we ran each model 200 times, using different 80% training and 20% test splits, and calculated the average relative importance of each ecological trait. To facilitate comparisons of measures of relative importance across BRT models, we normalized these measures to the variable of greatest importance (i.e., citation count).

### 2.4 Predicting viral reservoirs

**FIGURE 1:**
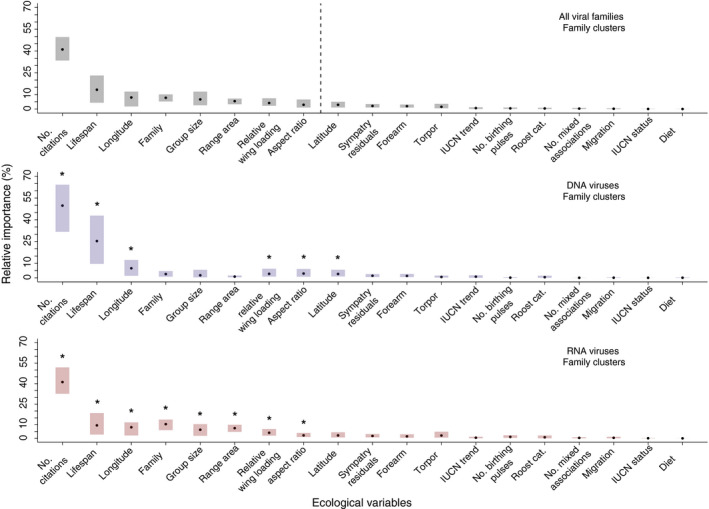
Relative importance of ecological traits from 200 BRT model runs. Points represent average relative importance across runs, while shaded bars represent the range within which 95% of values fall. In the upper panel, traits summing to ~90% of the average relative importance for models are separated by a dashed line. In the lower two panels, traits summing to 90% of the average relative importance for models are marked with asterisks. *Top Panel:* Relative importance measures for predicting total viral family diversity. *Middle Panel:* Relative importance measures for predicting DNA viral family diversity. *Bottom Panel:* Relative importance measures for predicting RNA viral family diversity

We used the predict function from the gbm package (Ridgeway, 2017), and our models for total and zoonotic viral family diversity to determine which of the zeros in our dataset (i.e., species not known to be reservoirs; *n* = 540) were likely to carry viruses. Given that citation count was the most influential predictor in all cases, using the BRT models presented above would result in bat species’ being predicted as viral reservoirs largely based on study effort, rather than any intrinsic traits. Further, simply removing citation count from the analyses would lead to an increase in the relative importance of those traits correlated with citation count (e.g., group size, life span) that may also be proxies for study effort in this dataset. To ensure that species were predicted as potential reservoirs based on their ecological traits and not because they were well studied or possessed the traits of well‐studied species, we built BRT models that excluded citation count and removed its effects from continuous variables. We regressed each predictor that was correlated with citation count (Figure A2) and had high relative importance in the total virus diversity model (Figure 1)—group size, latitude, longitude, life span, and range area—against citation count. We then built new BRT models using the residuals as predictors, along with the other raw variables. We could not correct for citation count in categorical variables (e.g., bat family, torpor use), but note that citation count may influence these predictors (Figures A6,A7) and discuss implications in the results.

Using residual predictors, we ran 200 BRT models with total viral family diversity as the response and different 80% training and 20% test splits of the data. For each run, we generated predictions for the number of viruses harbored by each bat species and averaged these values over the 200 runs (Appendix C). As in (Han et al., 2015, 2016), we looked specifically at bats with no known viruses (zeroes in our dataset) and identified the most likely reservoir species as those falling within the top 90th, 95th, or 99th percentile for predicted number of viruses (Appendix C). We also repeated this process for zoonotic viral families (Appendix C) and for RNA and DNA viral families (only 99th percentile predictions; Appendix C). For average measures of relative importance and predictive performance for BRT models that accounted for the influence of citations, see Figures A8–A11.

We mapped the geographic distribution of bat species (obtained from IUCN, 2017) to gain an understanding of the global distribution of potential viral carriers. In ArcGIS (*v. 10.1*; ESRI, 2010), we performed a raster overlay analysis to create five maps (resolution 0.1 × 0.1 decimal degrees): range overlap for all bat species, range overlap of confirmed viral carriers, range overlap of predicted viral carriers in the 90th percentile of model predictions for both total and zoonotic viral diversity, and range overlap of predicted viral carriers in the 95th percentile of model predictions for total viral diversity. We then divided each raster cell in our maps for range overlap of predicted viral carriers by the total bat biodiversity in that cell, to control for the fact that bat reservoirs are more likely to come from areas of higher overall bat diversity. This produced additional maps showing the proportion of bat species in any given area that are predicted viral carriers. In the main text, we present the range overlap and scaled proportional diversity map for species predicted to be viral carriers using the total viral diversity model (90th percentile). All other maps are in Appendix A (Figures A12–A14).

## 3 RESULTS

### 3.1 Ecological traits important for predicting viral diversity

Models for total viral family diversity in bats had high average predictive accuracy (pseudo‐*R* ^2^_test.average_ = 0.52; Figure A16; Table A3), comparable to prior work (Han et al., 2015, 2016). BRT models for RNA viral families had similar average predictive accuracy (pseudo‐*R* ^2^_test.average_ RNA = 0.46; Figure A16; Table A3). Models had lower accuracy when predicting DNA viral families (pseudo‐*R* ^2^_test.average_ DNA = 0.27; Figure A16; Table A3), likely due to their limited occurrence in the dataset (Figures A4,A5). Models also had lower accuracy when predicting zoonotic viral families (pseudo‐*R* ^2^_test.average_ zoonotic = 0.36; Figure A11).

**FIGURE 2:**
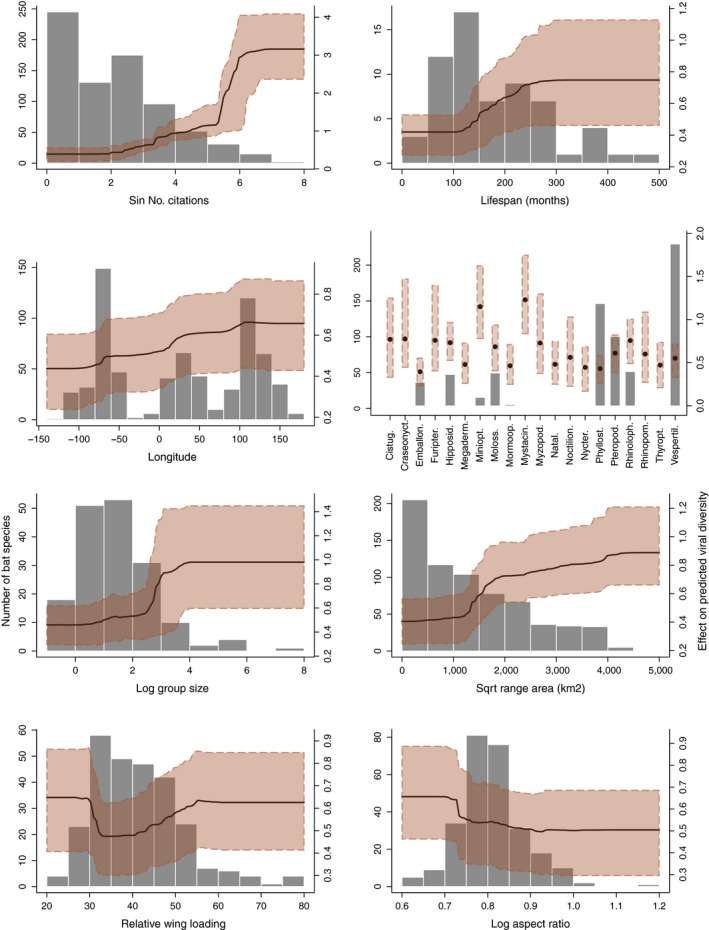
Partial dependence plots for ecological traits most important for predicting total viral family diversity. Black lines/points show the average effect of traits on predicted viral diversity from 200 BRT model runs, while red‐shaded regions represent the range within which 95% of values fall. Histograms and barplots showing the distribution of observed values for ecological traits are included in the background as gray bars

Traits important for predicting total viral family diversity included the following: citation count, life span, longitude of range midpoint, bat family, median group size, range area, relative wing loading, and aspect ratio (Figure 1). The relative importance of these eight traits in BRT models summed to ~90%. Averaged partial dependence plots for individual traits indicate that citation count, life span, longitude, and median group size had positive effects on predicted viral diversity (Figure 2), as expected (Table A2). Put another way, our BRT models predict that species that are better studied, longer‐lived, form larger social groups, and have larger geographic ranges east of the Prime Meridian carry the greatest number of viral families. For relative wing loading, both low and high values had a positive, albeit small, effect on predicted viral diversity, while increasing aspect ratio had a small, negative effect in BRT models (Figure 2). Variability around the partial dependence plots for these morphological traits makes it difficult to draw strong inferences (Figure 2). Models predicting the distribution of zoonotic viruses emphasized the same trait profile (i.e., longer‐lived bat species, widely distributed east of the prime meridian) as the total viral family diversity model (Figure A10).

**FIGURE 3:**
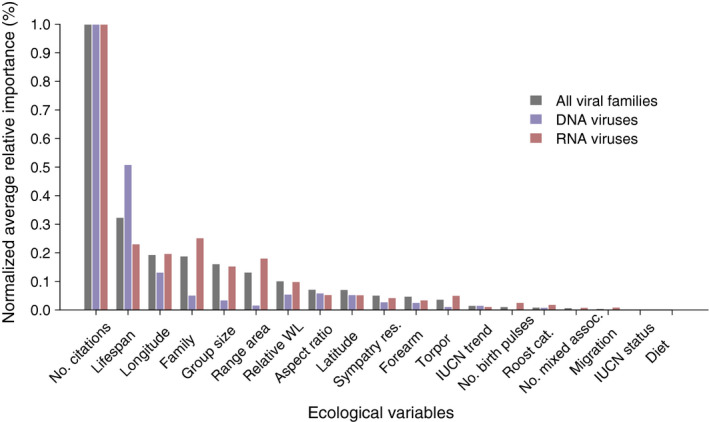
Average measures of relative importance from 200 BRT model runs (i.e., points from Figure 1), normalized with respect to the most important ecological variable in each model (number of citations in all cases). Gray, blue, and red bars are results from models predicting total, DNA, and RNA viral family diversity, respectively

The ecological traits summing to ~90% of the relative importance for models predicting RNA, DNA, and total viral family diversity were largely the same (Figure 1). However, the normalized measures (Figure 3) reveal shifts in the relative importance of traits for predicting the diversity of DNA, but not RNA viruses. For DNA viruses, life span was more important, while family, group size, and range area were less important than in models predicting total and RNA viral family diversity (Figure 3). Partial dependence plots indicate that the shape of the relationships between these traits and RNA or DNA viral diversity is similar to what is predicted for total and zoonotic viral diversity (Figures A17–A22).

### 3.2 Predicted viral reservoirs

To predict suspected viral carriers, we ran BRT models that excluded citation count as its own variable, but corrected for its influence on key predictors. We found that these residual (rather than raw) predictors resulted in few changes in the relative importance of traits (Figures A8,A10). Life span residuals, longitude residuals, relative wing loading, family membership, group size residuals, aspect ratio, and geographic range area residuals all remained within the top predictors for both total and zoonotic viral diversity (Figures A8,A10). However, torpor use, forearm length, and latitude were more important for prediction in the residual model for total viral family diversity (Figure A8), while torpor use was more important in the residual model for zoonotic viral diversity (Figure A10). Distance from the equator, forearm length, and hibernation all had positive effects on viral diversity (Figure A15). Since we were unable to remove the influence of citation count on hibernation (a categorical variable), this increase in relative importance may be driven by a correlation between hibernation and citation count (Figure A6). All other ecological traits had similar effects on predicted viral diversity as before (Figures A21,A22). Finally, the predictive power of the residual models was comparable to other models (Figures A9,A11).

Looking at bat species that harbor no viruses according to the dataset, and using the top 90th percentile of predictions from the total viral family diversity models, we predict 55 species from 13 bat families may be undetected viral carriers (Figure A15). Using the 95th or 99th percentile as a cutoff, 27 and six species are predicted to be undetected carriers, respectively (Appendix C). For zoonotic viral families, we predict 54, 28, and six species may be undetected carriers using the 90th, 95th, and 99th percentiles, respectively (Appendix C). For both total and zoonotic viral richness, the same six species, *Asellia tridens*, *Barbastella barbastellus, Coelops frithii, Myotis grisescens, Phyllostomus hastatus,* and *Pteropus rodricensis*, were included in the 99th percentile of model predictions. Species identified in the 95th percentile of the zoonotic model were also the same as those identified by the total viral family diversity model (Appendix C); however, there were minimal shifts in the species predicted as zoonotic reservoirs in 90th percentile (Appendix C). In Appendix C, we also list species in the top 99th percentile of model predictions for only RNA or DNA viral families. Species predicted to be carriers of RNA viral families are the same six listed above. Conversely, for DNA viruses, the six species in the 99th percentile include three of the above bats (*Myotis grisescens*, *Pteropus rodricensis*, and *Barbastella barbastellus*), in addition to three other species (*Hipposideros fulvus*, *Haplonycteris fischeri*, and *Myotis sodalis*)*.*

**FIGURE 4:**
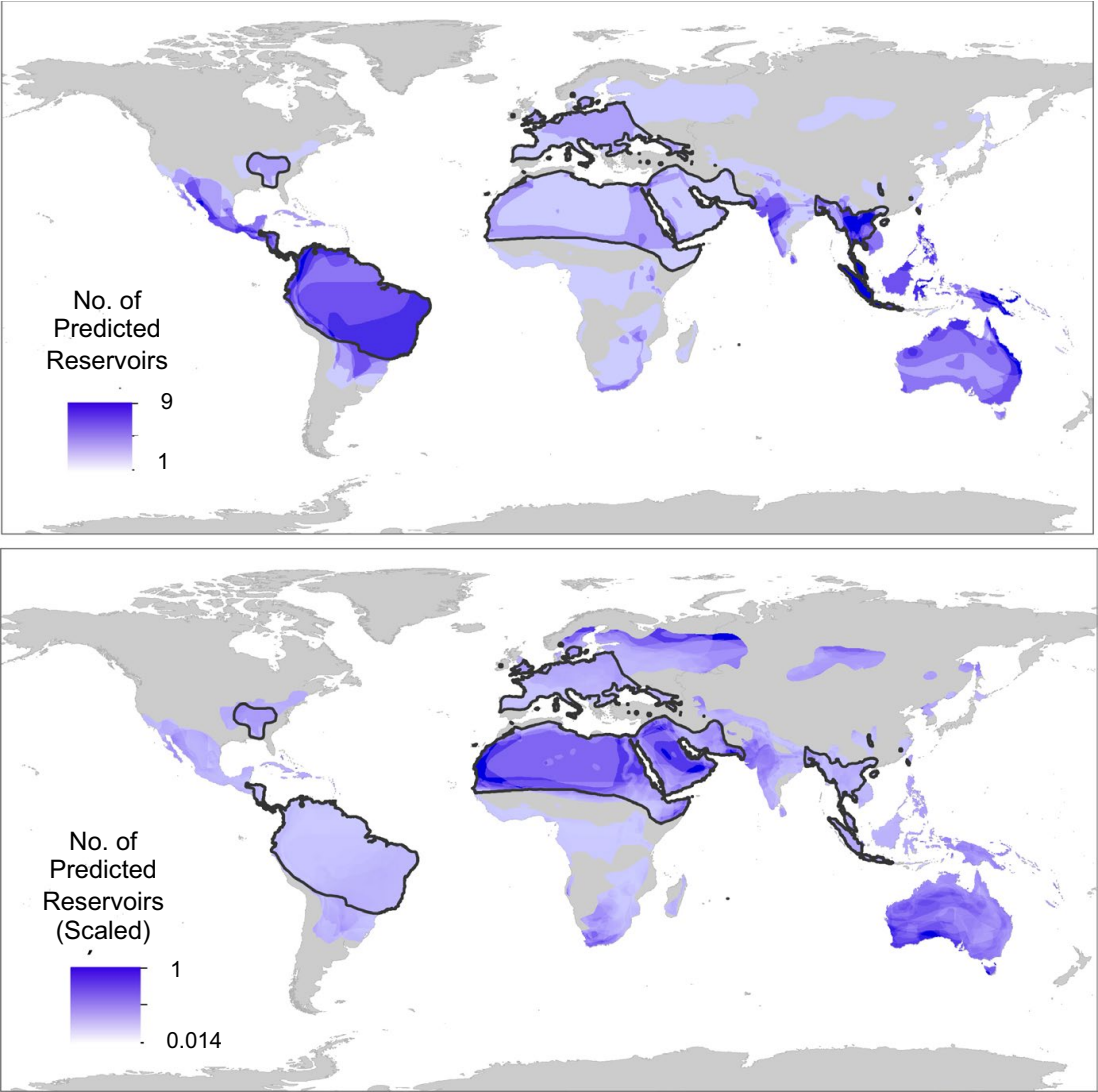
Global distribution of predicted viral carriers. *Top Panel:* Overlay map of bat species predicted to be undetected viral carriers (top 90th percentile of model predictions, averaged over 200 runs, for number of viral families hosted by bat species that are considered noncarriers in the dataset). *Bottom Panel:* Same as top panel, but scaled to the total bat diversity in a given area, i.e., darker regions represent areas where a greater proportion of the local bat species are predicted to be undetected viral carriers. Black outlines highlight the ranges of the six bat species within the 99th percentile of model predictions

Geographically, predicted reservoir species are concentrated in Southeast Asia and South America (Figure 4, top), as would be expected given the global distribution of bat biodiversity and confirmed reservoirs (Figure A12). There were also hotspots of predicted reservoirs along the northern and eastern coasts of Australia (Figure 4, top). Scaling the predicted reservoir map by total bat biodiversity indicates that predicted reservoirs account for a greater proportion of local bat diversity in Northern Africa, the Middle East, Northern Europe, and southwestern tip of Australia (Figure 4, bottom). While we present results for predicted reservoirs based on our total viral family diversity models in the main text, results from the model using only zoonotic viral families are the same (see Figure A14).

## 4 DISCUSSION

Bats are carriers for a vast number of viruses, many zoonotic (Wang & Anderson, 2019). This necessitates a deeper understanding of drivers of viral diversity and preemptive identification of reservoirs. Here, we develop a predictive model that distinguishes reservoir species from nonreservoirs, and further delineates those reservoirs into those harboring zoonotic viruses, RNA or DNA viruses. We confirm the importance of traits previously identified (e.g., geographic range size and group size) as predictors of viral diversity in small samplings of bat species or for single viral families, but also highlight additional predictors of viral diversity (e.g., longitude, life span). Specifically, we show that longer‐lived bat species that form larger social groups and are more widely distributed east of the Prime Meridian, host the greatest diversity of viruses. We also find that there are few differences in traits important for predicting the diversity of viruses with different genome structures (Figure 1): life span had a greater influence on DNA compared to RNA viruses (Figure 3), while group size and family are more important predictors for RNA viruses (Figure 3). Finally, we identify species that, although not currently classified as reservoirs, are likely to carry viruses. Predicted reservoir species are concentrated in Southeast Asia and South America (Figure 4, top), but account for a greater proportion of local bat diversity in northern Africa, Europe, and the Middle East (Figure 4, bottom).

As with previous work in smaller subsets of bat species (e.g., Guy et al., 2019; Webber et al., 2017), we find that geographic range size and group size have positive associations with predicted viral diversity (Figure 2). Large group sizes and geographic ranges may indeed be universal predictors of parasite richness (Kamiya et al., 2014), corresponding to increased opportunities for interactions between con‐ and hetero‐specifics that may facilitate pathogen transmission (Altizer et al., 2003; Altizer, Bartel, & Han, 2011; Patterson & Ruckstuhl, 2013). This may be particularly true for bats, as many species with large ranges migrate or hibernate, increasing potential exposure to pathogens as they move through different regions or use seasonal roosting sites that differ in population structure and species composition (Kunz, 1982). Despite this, species sympatry was not important for prediction. This result, in line with (Webber et al., 2017), but in contrast to other work (e.g., Luis et al., 2013), may be explained by the fact that we use the residuals of species’ sympatry (regressing sympatry on geographic range size), controlling for the fact that widely distributed bats are more likely to experience range overlap. We thus infer that range size is a more important driver of viral diversity than sympatry per se*.*

Species citation count aside, life span was the most important predictor of viral diversity (Figure 1). Our models suggest that longer‐lived species carry a greater number of viruses, likely due to increased exposure. Species with slow life histories (i.e., increased longevity, larger‐bodied, longer juvenile development) are expected to accumulate more infections in their lifetimes (Poulin & Morand, 2004), favoring selection for costly immune defenses (Miller, White, & Boots, 2007). Slow‐lived species also tolerate and limit infection‐induced pathology better than species with faster life histories (Johnson et al., 2012). Comparing across mammals, bats fall at the slow end of the fast/slow life‐history continuum (Barclay & Harder, 2003). Despite small body sizes and high metabolic rates, their longevity suggests efficient mechanisms for dealing with oxidative damage (Brook & Dobson, 2015; Munshi‐South & Wilkinson, 2010) that may be co‐opted to help bats tolerate viral infections (Brook & Dobson, 2015). While their slow life histories suggest that bats, in general, are better equipped to deal with viruses than shorter‐lived mammals, our models suggest that among species, time available (i.e., life span) for potential exposure predicts the diversity of acquired infections.

While life span was important for predicting overall viral diversity, it had even greater importance for predicting the diversity of DNA viruses (Figure 3). Compared to RNA viruses, DNA viruses are thought to more frequently codiverge with their hosts (Holmes, 2009), which may be a function of longer infection durations (Geoghegan, Duchêne, & Holmes, 2017; Villarreal et al., 2000). DNA viruses that cause chronic infections might have higher fitness in longer‐lived individuals, through increased transmission opportunities (Villarreal et al., 2000). Conversely, many RNA viruses have short infection durations (Holmes, 2009; Villarreal et al., 2000). The acute nature of these infections may explain why group size is more important in RNA than DNA models (Figure 3): high contact rates could sustain transmission of short‐lived infections in populations, leading to episodic pulses of viral shedding (Plowright et al., 2016). Additionally, RNA viruses experience cross‐species transmission more frequently (Geoghegan et al., 2017), and cross‐species emergence is constrained by host relatedness (Streicker, 2013), which could explain why bat family had higher relative importance in RNA models (Figure 3). We note that RNA viruses dominate our dataset, explaining similarities in inferences from models predicting total or RNA viral family diversity. Occurrences of DNA viruses are limited, making strong inferences from model predictions more difficult.

Consistent with current patterns of bat zoonotic emergence (e.g., SARS in China, Hendra in Australia, Nipah in Malaysia; reviewed in Wang & Anderson, 2019), our models predict that species east of the Prime Meridian carry more viruses (Figure 2). In line with identified hotspots for zoonotic disease emergence (Morse et al., 2012), our models also predict a concentration of potential bat reservoirs in Southeast Asia. We also find a hotspot of potential reservoirs in the Neotropics (Figure 4). While this matches patterns of confirmed viral carriers (Figure A12), and the expectation that host diversity fosters parasite diversity (Hechinger & Lafferty, 2005), zoonotic disease emergence has disproportionately been in the eastern Hemisphere (Jones et al., 2008). This highlights that reservoir distribution is not necessarily synonymous with human disease risk, as a myriad of factors (e.g., human population density, bush meat hunting) influence viral sharing between bats and humans (Brierley, Jones, Vonhof, Olival, & Daszak, 2016). Future work incorporating these risk correlates with maps of predicted reservoirs could help guide viral surveillance efforts. Global regions identified as hotspots by our scaled maps provide interesting targets since, in these areas, predicted viral carriers account for a greater proportion of local bat fauna.

The methodology we apply here was previously used to examine filovirus reservoir status in bats (Han et al., 2016) and not surprisingly some of our inferences are consistent with that analysis. However, filoviruses comprise a small part of our dataset (1 of 26 viral families), so naturally we also find differences: for example, in our analysis faster reproductive rates are not correlated with reservoir status (as in Han et al., 2016). We further build on the methodology of Han et al. (2016, 2015) by explicitly testing several model assumptions (e.g., effect of zero inflation, importance of phylogenetic grouping; Appendix B) and accounting for inherent variability between model runs by drawing conclusions from multiple iterations. Lastly, given the importance of study effort in previous work (e.g., Guy et al., 2019), here we attempt to remove this effect before generating predictions to avoid species being predicted as reservoirs simply because they are well studied or possess the traits of well‐studied species.

Finally, we identify six bat species most likely to be zoonotic viral carriers: *Asellia tridens*, *Barbastella barbastellus, Coelops frithii, Myotis grisescens, Phyllostomus hastatus,* and *Pteropus rodricensis*. While there was no viral sequence data for these species in DBatVir (Chen et al., 2014), there is evidence that three of these bats (*B. barbastellus, M. grisescens, P. hastatus*) are viral reservoirs (see Luis et al., 2013), suggesting our models are predicting well. While this highlights the challenges of data curation for macroecological studies, supplementary analyses indicate that additional viral information does not change model inferences (Appendix B.4).

The three species that are consistently identified as likely zoonotic viral carriers—*A. tridens, C. frithii, P. rodricensis*—vary substantially in their biology, highlighting the power of machine learning approaches for identifying outcomes that may not have been expected a priori. The first two species are insectivorous hipposiderids, but while *A. tridens* ranges throughout the Middle East and northern Africa (Amichai, Levin, Kronfeld‐Schor, Roll, & Yom‐Tov, 2013), *C. frithii* is a southeast Asian species (Ho, Fang, Chou, Cheng, & Chang, 2013) about which little is known (Bates, Bumrungsri, Francis, Csorba, & Molur, 2008). Conversely, *P. rodricensis* is a large, Old World fruit bat (Pterpodidae), endemic to the smallest of the Mascarene Islands (Powell & Wehnelt, 2003). *C. frithii* and *A. tridens* are considered of least concern by the IUCN (Bates et al., 2008; Monadjem et al., 2017), but *P. rodricensis* is endangered (Tatayah, Jhangeer‐Khan, Bégué, & Jones, 2017). This highlights the challenge of careful communication of risk from studies like ours that identify potential reservoir species. While we identify bat species that are likely to harbor viruses, many of these species provide critical ecosystem services including pollination, seed dispersal, and insect control (Kunz, Torrez, Bauer, Lobova, & Fleming, 2011). We are cognizant of the impact negative framings may have on bat conservation (López‐Baucells, Rocha, & Fernández‐Llamazares, 2018) and hope that our results motivate not only viral surveillance efforts, but also the development of strategies that minimize disease risk to humans while simultaneously considering bat conservation.

## 5 COMPETING INTERESTS

The authors declare no competing interests.

## AUTHOR CONTRIBUTIONS

**Cylita Guy:** Conceptualization (equal); Data curation (lead); Formal analysis (lead); Methodology (equal); Visualization (lead); Writing‐original draft (lead); Writing‐review & editing (equal). **John Ratcliffe:** Conceptualization (equal); Funding acquisition (equal); Supervision (equal); Writing‐review & editing (equal). **Nicole Mideo:** Conceptualization (equal); Funding acquisition (equal); Methodology (equal); Resources (lead); Supervision (equal); Writing‐review & editing (equal).

## Supporting information

Appendix A

Appendix B

Appendix C

## Data Availability

Data and samples of code for analyses can be found on Dryad: https://datadryad.org/stash/share/7UhUQlyGOm1FYN31s6FsX7rk‐gq_iZk8kYQBoESEmzM
