# Supplementary material for: The influence of bat ecology on viral diversity and reservoir status
Source: Ecol Evol. 2020 May 8;10(12):5748–58. doi: 10.1002/ece3.6315 (PMC7319232; doi:10.1002/ece3.6315)
Supplement: Supplementary file 1 — Appendix A [file ECE3-10-5748-s001.pdf]

Table A1. Bat ecological traits used in boosted regression tree models. Numbers and percentages of bat species – total and those carrying viruses – for which each variable could be collected are included. These values are for the full dataset (n=812 species in the Shi and Rabosky (2015) phylogeny).

Ecological Trait	Data coverage for bats carrying viruses (215 species)	Data coverage for all bats (812 species)	Trait Description	Units
Forearm Length	191 (88.8%)	632 (77.8%)	Total length from elbow to wrist of adult (or age unspecified) live, freshly-killed, or museum specimens using captive, wild, provisioned or unspecified populations; males, females, or sex unspecified individuals. All measures of central tendency, in all localities. Values log transformed for analysis.	mm
Relative Wing Loading	122 (56.7%)	269 (33.1%)	Weight (in kg) times acceleration due to gravity divided by the wing area (m^2), standardized by mass of adult (or age unspecified) to the power of 1/3 live, freshly killed, or museum specimens using captive, wild, provisioned or unspecified populations; males, females, or sex unspecified individuals. All measures of central tendency, in all localities.	N/A
Aspect Ratio	115 (53.4%)	267 (32.9%)	Square of the wingspan (in m^2) divided by the wing area of adult (or age unspecified) live, freshly killed, or museum specimens using captive, wild, provisioned or unspecified populations; males, females, or sex unspecified individuals. All measures of central tendency, in all localities. Values log transformed for analysis.	N/A
Lifespan	47 (21.7%)	62 (7.6%)	Maximum age of adults measured through direct observation, capture-recapture, or projected from physical wear using males or females from captive or wild populations, in all localities.	months
Migration	76 (35.4%)	121 (14.9%)	Categorization of migration where: N = no evidence for migration and Y = evidence for migration (regional and long distance).	N/A
Diet	211 (98.1%)	796 (98%)	Categorization of diet where: Pred = predatory species (consuming insects, vertebrates, *etc*.) or Phyto = phytophagous species (consuming nectar, fruit, plant matter, *etc*.)	N/A
Torpor Expression	108 (50.2%)	176 (21.7%)	Categorization of torpor use where NT = no evidence of torpor, T = some use of torpor, H = true hibernation	N/A
Median Aggregation Size	79 (36.7%)	170 (20.9%)	Number of individuals (adults, juveniles, or unspecified) observed in a species aggregation in wild populations. All measures of central tendency, in all localities, calculated using provided ranges of individuals in groups (*i.e*., min – max was provided, but not average) where unavailable. Values log transformed for analysis.	No. of individuals
Roost Type	153 (71.2%)	429 (52.8%)	Categorization of primary structures used for roosting for the majority of the season where: CV = cave/crevice, C = cavity, F = foliage/external, G = generalist/anthropogenic structures.	N/A

Predictor	% Coverage Viral Bats (215 species)	% Coverage All Bats (812 species)	Description	Units
Number of Mixed Species Associations	63 (29.3%)	133 (16.4%)	Number of other bat species with which a focal species has been found in association in a roost. Values inverse hyperbolic sine transformed for analysis.	No. of species
Birthing Pulses	126 (58.6%)	258 (31.8%)	Number of peaks in birthing periods throughout the year as determined from wild populations.	No. of birthing peaks
IUCN Status	203 (94.4%)	729 (89.4%)	IUCN conservation status where: LC = least concern, NT= near threatened, V= vulnerable, E = Endangered, CE = critically endangered	N/A
IUCN Population Trend	203 (94.4%)	729 (89.4%)	IUCN population trend where: D = decreasing, I = increasing, S= stable, U = unknown	N/A
Latitude Range Midpoint	183 (94.4%)	680 (89.4%)	Latitude of central point of species geographic range polygon (taken from the IUCN), calculated to minimize the Euclidian distance to all edges in the distribution (decimal degrees).	dd
Longitude Range Midpoint	183 (94.4%)	680 (89.4%)	Longitude of central point of species geographic range polygon (taken from the IUCN), calculated to minimize the Euclidian distance to all edges in the distribution (decimal degrees).	dd
Geographic Range Area	183 (94.4%)	680 (89.4%)	Measure of species geographic range area, made using IUCN range area polygons. Values square root transformed for analysis.	km^2
Residuals of Species Sympatry	183 (94.4%)	680 (89.4%)	Residuals taken from regression of square root transformed range area on species sympatry. Species sympatry is a measure of the number of species whose geographic range overlaps with the range of a focal bat species.	N/A
Number of Citations	215 (100%)	812 (100%)	Number of publications for each bat species binomial name returned on Web of Science. Values inverse hyperbolic sine transformed for analysis.	No. of citations

Table A2. Predicted relationships between bat ecological traits and viral richness. Bat traits were either predicted to have a positive (+) or (-) relationship with viral diversity.

Ecological Trait	Predicted Relationship with Viral Richness	Justification
Forearm Length	+	In bats, forearm length is used to assess body size. Larger hosts may provide parasites with more resources, or encounter parasites at greater rates through ingestion and contact (Lindenfors et al. 2007, Kamiya et al. 2014).
Relative Wing Loading & Aspect Ratio	-	RWL and AR serve as rough proxies for bat ecological niches (Norberg and Rayner 1987) and capture variation in daily activity patterns. The high body temperatures and metabolic rates associated with bat flight could play a role in pathogen control (O'Shea et al. 2014), given that immune responses are thought to be potentiated at high temperatures (*i.e.* fever; Hasday et al. 2000, Blatteis 2003). Species with wings designed for long periods of sustained flight (aerial insectivores; high AR, high RWL), leading to sustained increases in body temperature, may host a lower diversity of pathogens, owing to faster clearance and lower probability of detection.
Lifespan	+	Long lifespans provide more time for exposure to parasites (Poulin and Morand 2004).
Diet	Phytophagy > Predatory	Shared food resources (*i.e.* partially consumed pieces of fruit) may become contaminated with viruses (Dobson 2005), increasing opportunities for viral transmission between individuals. Frugivorous and nectivorous species (*i.e.* phytophagus) may therefore harbor greater pathogen diversity than predatory species.
Torpor Expression	-	Torpor use is predicted to correlate negatively with viral diversity, since viral replication and host contact rates will be reduced in hibernation (Luis et al. 2013, Guy et al. 2019), potentially limiting opportunities for viral transmission and leading to lower pathogen diversity.
Median Aggregation Size	+	Contact rates are likely to increase in larger groups, facilitating transmission (Calisher et al. 2006, Luis et al. 2015, Webber et al. 2017).
Migration	+	Migratory species may move through a greater diversity of habitats as they use different seasonal roosting sites, increasing potential exposure to pathogens.

**Ecological Trait**	**Predicted Relationship with Viral Richness**	**Justification**
Roost Type	Cave/Crevice, Cavity, Anthropogenic > Foliage	Environmental conditions may vary between roost types, e.g., temperature and humidity tend to be higher in cavity/cave roosts. Given that temperature and humidity are thought to play a role in the environmental persistence of some viruses (Sooryanarain and Elankumaran 2015), transmission and maintenance of viral pathogens may be more likely in cave and cavity roosts, compared to foliage roosts.
Number of Mixed Species Associations	+	Bats co-roosting in the same structures may interact, facilitating interspecies pathogen transmission.
Birthing Pulses	+	The increase in susceptible hosts and contact rates after birthing periods is necessary for the persistence of some viruses in bat populations (Hayman 2015). Species with a greater number of birthing pulses throughout the year may be better able to sustain viral transmission.
IUCN Status & Population Trend	+	Threatened, declining, and fragmented populations may be more susceptible to pathogen infection due to stress, reduced immune functioning, and poorer nutritional status (see Turmelle and Olival 2009 for discussion).
Latitude Range Midpoint	-	While conflicting evidence exists (Willig et al. 2003, Kamiya et al. 2014), we expect viral richness to increase towards the equator, similar to the latitudinal patterns of species richness observed in other groups (Willig et al. 2003)
Longitude Range Midpoint	+	No species of bat is transoceanic. To control for this natural geographic barrier, we include longitude to separate old from new world species. Further, zoonotic emergence has occurred disproportionally in the eastern hemisphere (Jones *et al.* 2008) and so there may be longitudinal variation in viral transmission between species.
Geographic Range Area	+	Wide-ranging species may encounter a greater diversity of habitat types or a greater number of species, increasing opportunities for parasite exposure and transmission (Poulin and Morand 2004, Lindenfors et al. 2007, Luis et al. 2013).
Residuals of Species Sympatry	+	For a given range size, species experiencing range overlap with a greater number of other bat species may have greater opportunities for contacts that facilitate pathogen transmission (Luis et al. 2013, Olival et al. 2017, Guy et al. 2019)
Number of Citations	+	Included to help control for sampling bias. Previous literature indicates parasite diversity scales positively with study effort (*e.g.*, Lindenfors et al. 2007, Luis et al. 2013)

Table A3. Representative boosted regression tree model runs for total virus family diversity and the diversity of RNA and DNA virus families. Results are based on single BRT models, rather than 200 iterations. Optimized values for shrinkage, interaction depth, and number of trees are included, along with measures of model performance.

	All Viral Families		DNA Viral Families		RNA Viral Families	
**Interaction Depth**	4		3		3	
**Shrinkage**	0.0001		0.0001		0.001	
**Total Trees**	60000		30000		10000	
**Best iteration**	54857		23263		7865	
**Pseudo R^2 (train)**	0.82		0.53		0.79	
**Pseudo R^2 (test)**	0.57		0.32		0.53	
	**Predictors**	**Rel.imp**	**Predictors**	**Rel.imp**	**Predictors**	**Rel.imp**
	Citations	36.26	Citations	46.77	Citations	39.41
	Lifespan	13.71	Lifespan	31.46	Longitude	10.24
	Family	9.03	Longitude	5.25	Family	10.10
	Group Size	8.33	RWL	3.41	Range Area	7.66
	Longitude	8.25	Family	2.89	Lifespan	7.17
	Range Area	5.89	Latitude	1.95	RWL	4.87
	RWL	4.51	Residuals	1.84	Residuals	3.23
	AR	3.41	AR	1.42	AR	3.11
	Latitude	3.06	Group Size	1.38	Group Size	2.98
	Forearm	2.39	Range Area	1.12	Latitude	2.67
	Sympatry	1.82	Forearm	0.94	Roost	1.49
	Torpor	1.51	Trend	0.56	Torpor	1.40
	Pulse	0.48	Roost	0.46	Forearm	1.29
	Roost	0.47	Torpor	0.35	Pulse	1.19
	Trend	0.45	Pulse	0.09	Mixed Group	1.17
	Mixed Group	0.24	Migration	0.06	Trend	1.14
	Migration	0.12	Diet_2	0.02	Migration	0.82
	Status	0.04	Status	0.01	Status	0.05
	Diet_2	0.03	Mixed Group	0.01	Diet_2	0.01

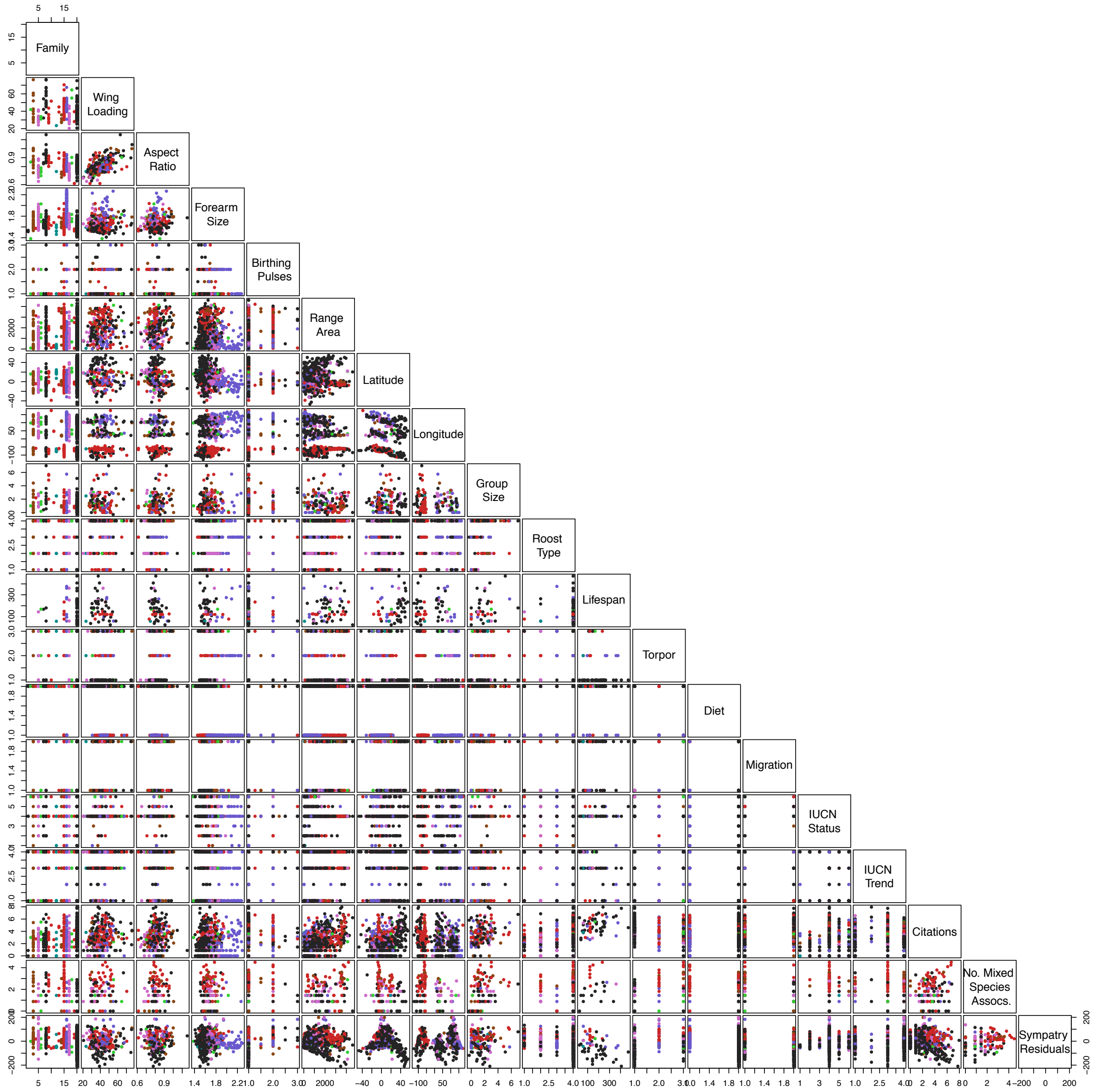

Figure A1. Pairwise plots of all bat ecological traits included in BRT models. Colors of points represent separation of species into the 56MYA phylogenetic clusters.

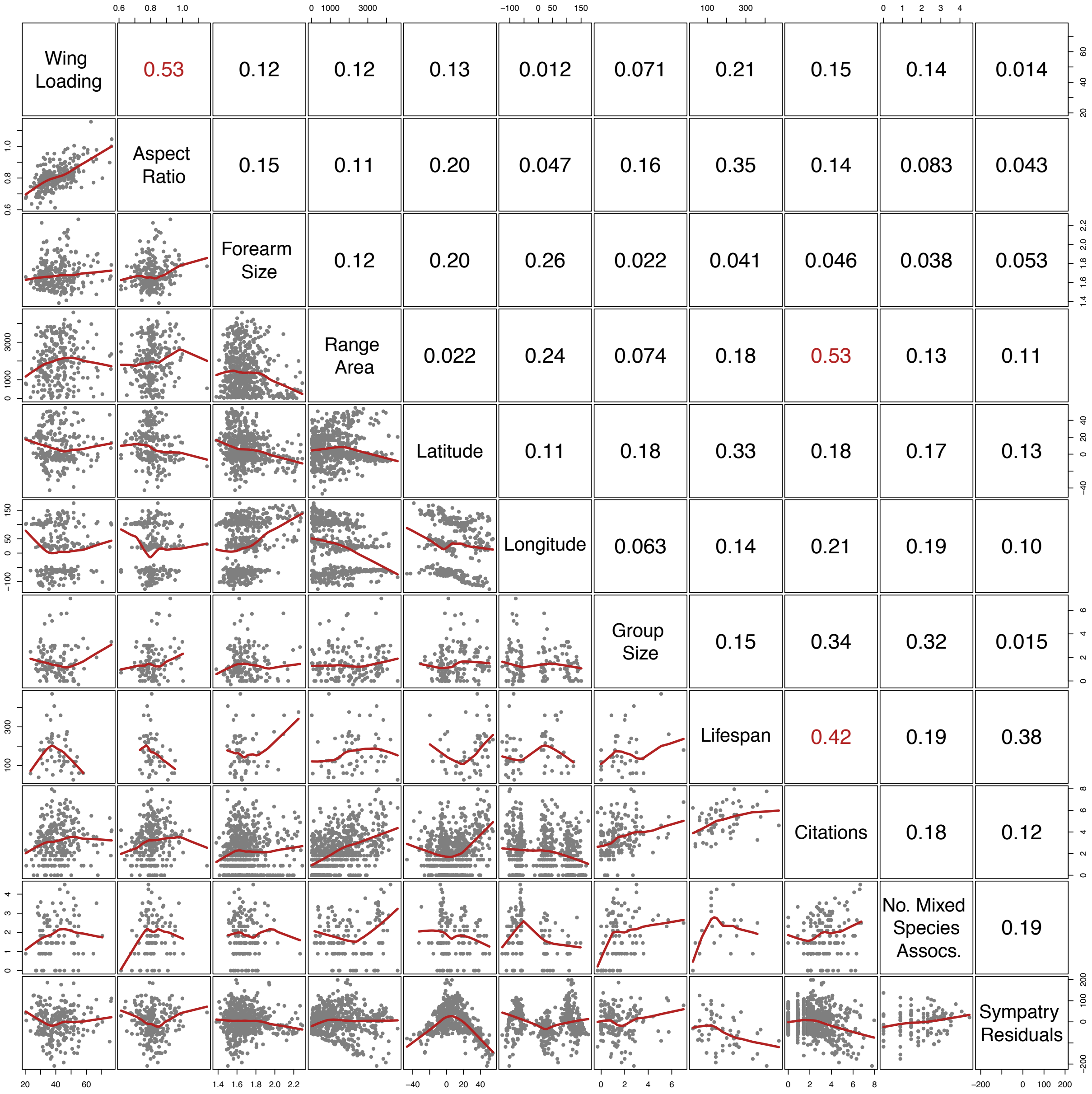

Figure A2. Continuous ecological predictors included in BRT models. Correlation coefficients are displayed in the upper right triangle. All coefficients where r > 0.4 are displayed in red. The lower left triangle contains pairwise plots of traits. Lines of best fit from a locally-weighted polynomial regression smoother (LOWESS) are shown in red. Note, citation count is correlated with both range area and lifespan and appears to have non-linear relationship with latitude, where bats further from the equator are better studied.

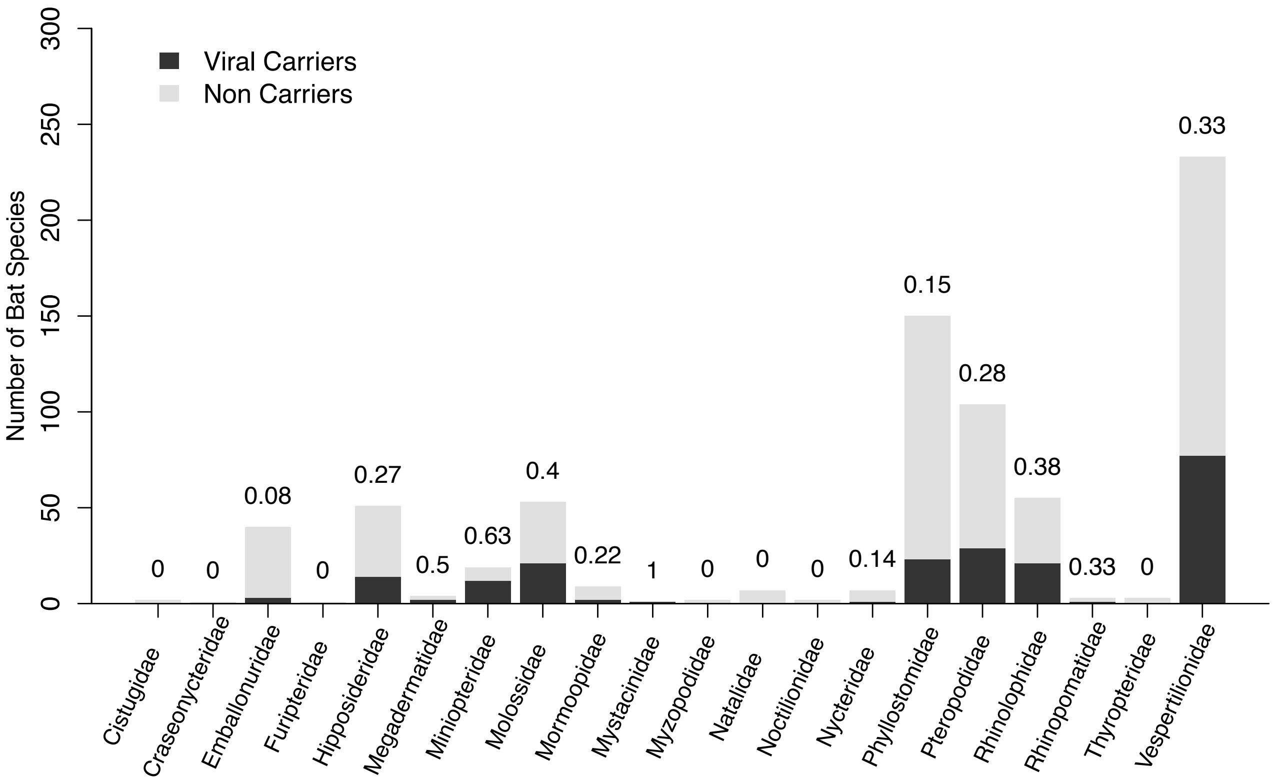

Figure A3. Distribution of bats in our dataset and confirmed viral carriers across bat families. Height of the bar indicates the number of species of each family included in the species' trait dataset. Dark gray portions of bars represent confirmed viral carriers, while light gray portions represent species considered to be non-carriers (*i.e.*, zeros) in our analysis. Proportion of viral carriers in each bat family is displayed numerically on the top of each bar.

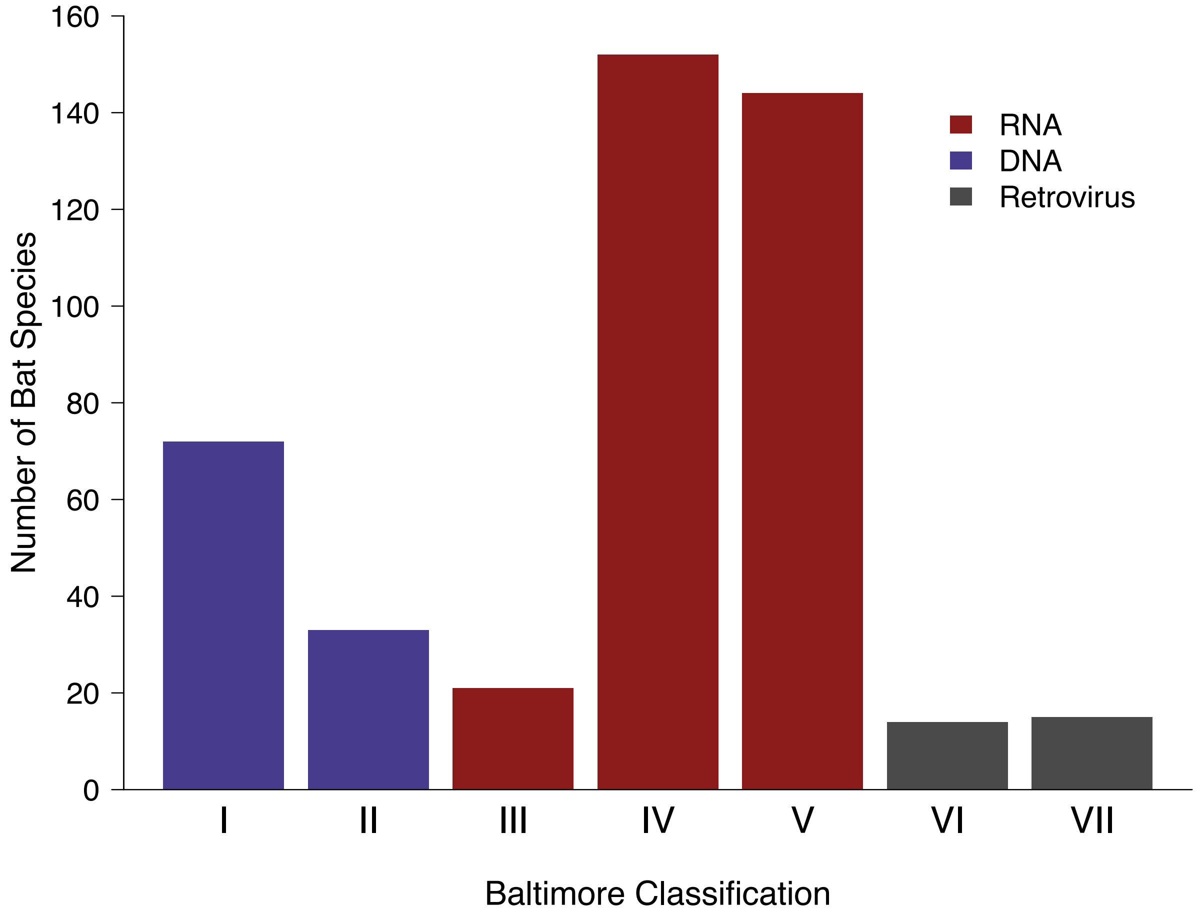

Figure A4. Number of bat species that have been reported to carry viral families of the seven Baltimore classification categories. Bars have been color coded to distinguish DNA, RNA, and retroviruses. Specific features of the Baltimore classes are as follows: (I) double-stranded DNA viruses, (II) single-stranded DNA viruses, (III) double-stranded RNA viruses, (IV) positive-sense single-stranded RNA viruses, (V) negative-sense single stranded RNA viruses, (VI) positive-sense single-stranded RNA retroviruses (DNA intermediate in replication), and (VII) double-stranded DNA retroviruses (RNA intermediate in replication).

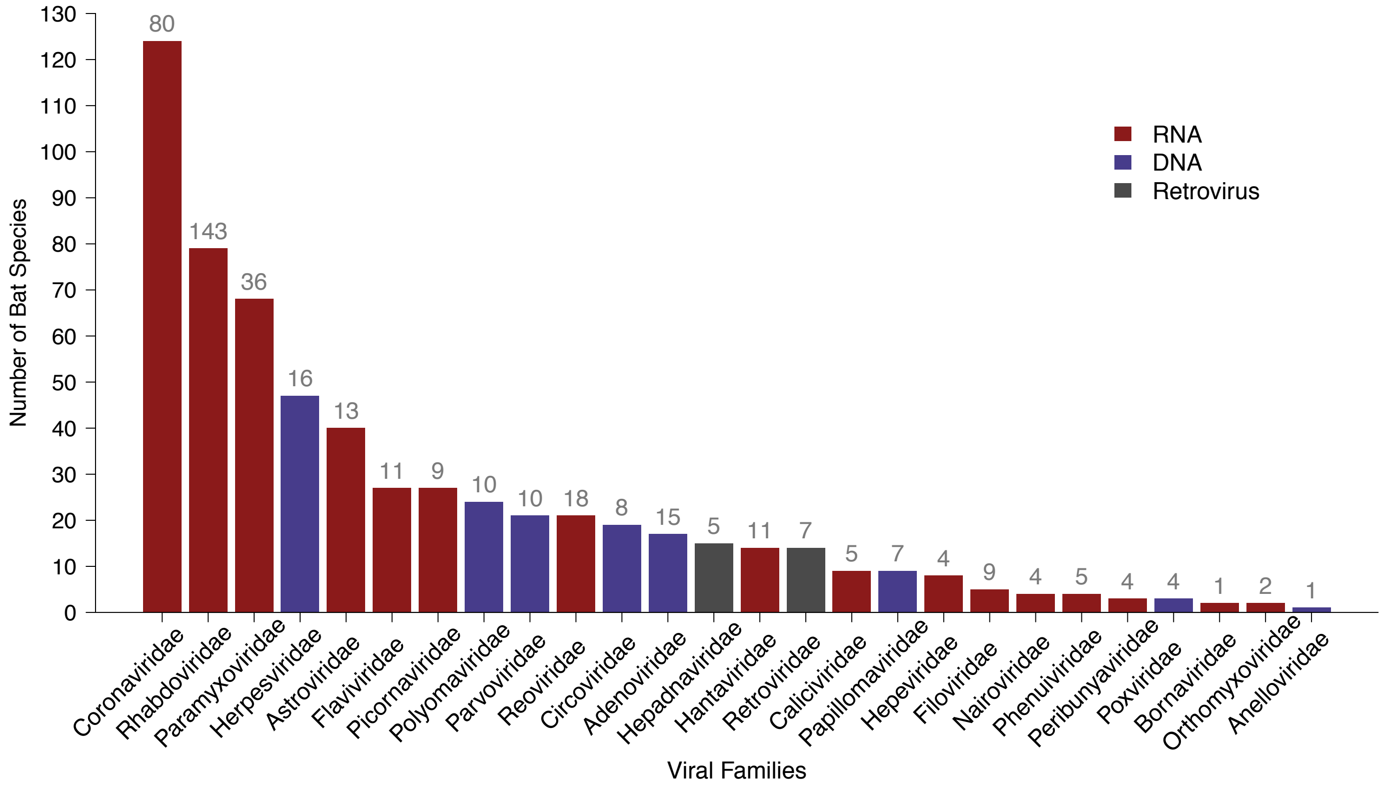

Figure A5. Number of bat species that have been reported to carry the viral families in our viral dataset. Bars have been color coded to distinguish DNA, RNA, and retroviruses. Numbers on top of bars indicate the number of unique publications for each viral family in the viral dataset.

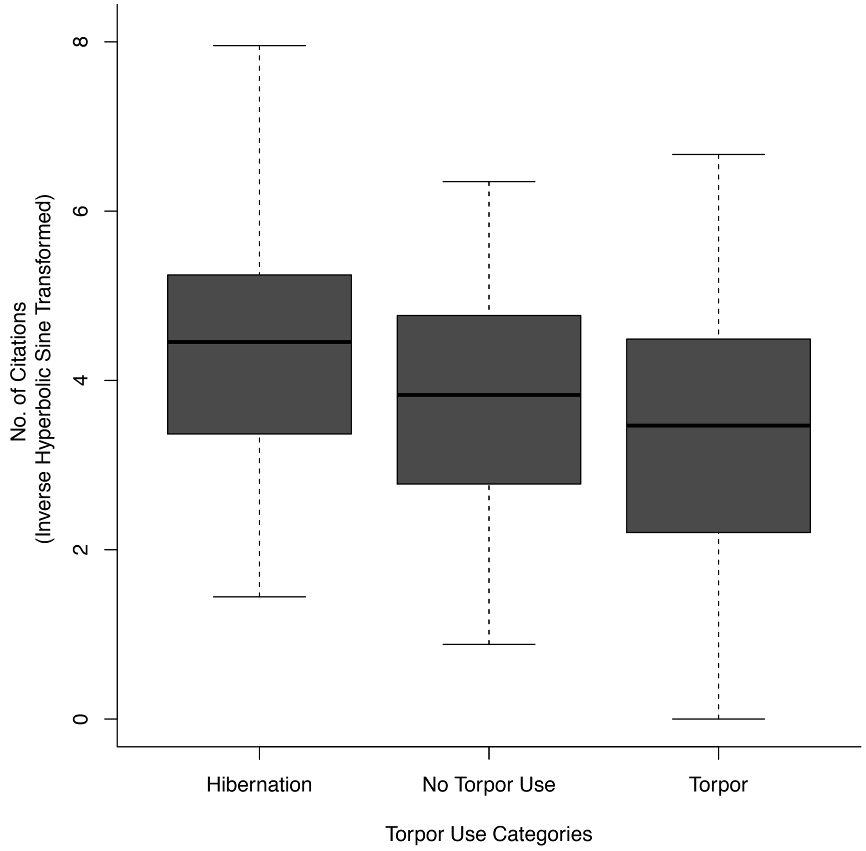

Figure A6. Boxplot showing distribution of citation count for each of the three torpor use categories. Results of a simulation based phylogenetically controlled ANOVA (performed using the phylANOVA function in phytools *v.0.6-44*, Revell 2012) indicates no significant difference in citation count between the three torpor categories ($F_{2,173}$=7.8, p=0.284). We also performed a non-phylogenetically controlled ANOVA (given that BRTs do not apply a phylogenetic correction) which indicated a significant difference in citation count between the three torpor categories ($F_{2,173}$=7.08, p<0.001). A Tukey's HSD test indicated that hibernators had significantly more citations (4.4±1.4SD, n=76) than both daily torpor users (3.3±1.7SD, n=52, p_{adj}<0.001) and non-torpor users (3.6±1.5SD, n=48, p_{adj}=0.03).

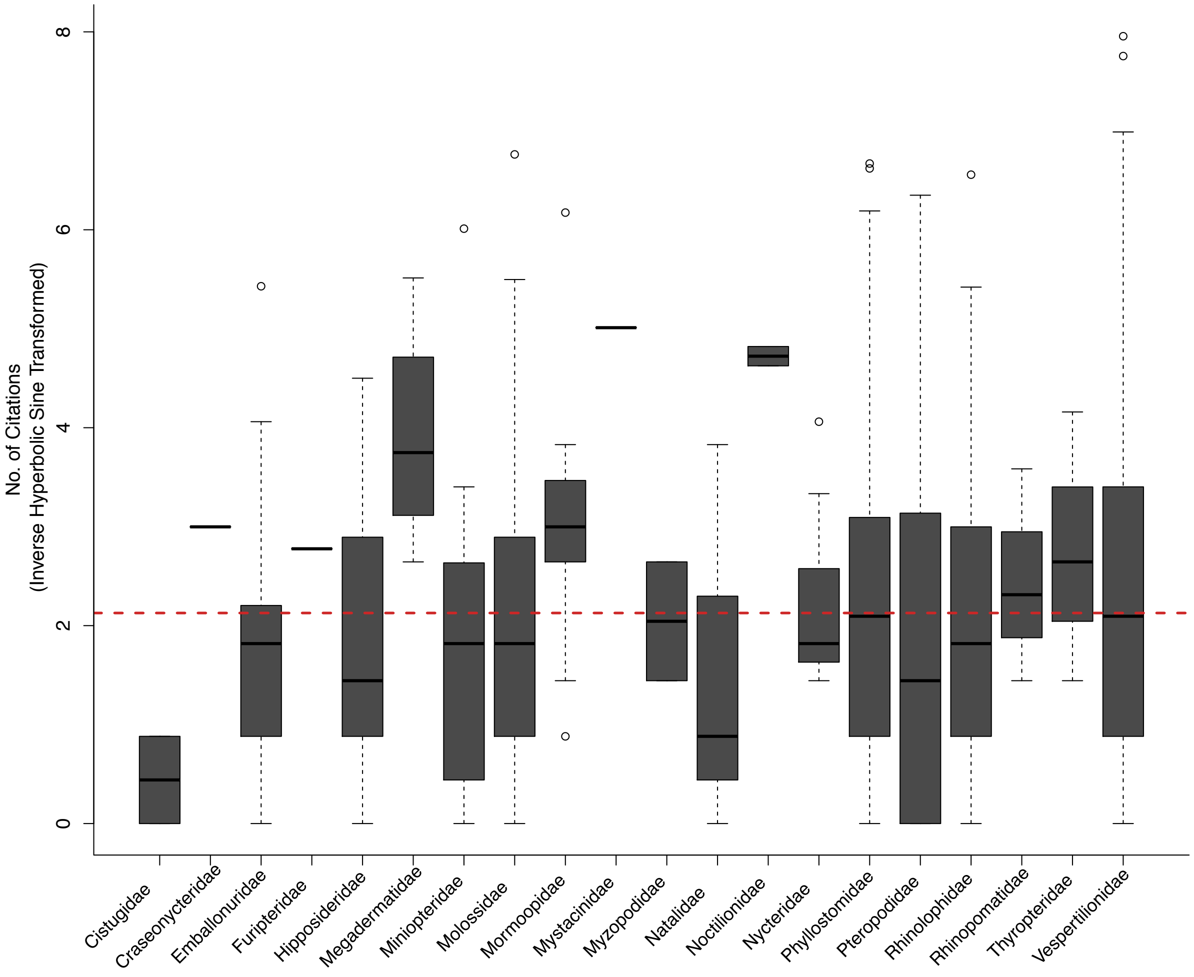

Figure A7. Boxplot showing distribution of citation count for each bat family included in the dataset (n=20). The red line represents average citation count across the dataset. Results of a simulation based phylogenetically controlled ANOVA (performed using the phylANOVA function in phytools *v.0.6-44*, Revell 2012) indicate no significant differences in citation count between bat families ($F_{19,792}$=1.64, p=1). We also performed a non-phylogenetically controlled Kruskal-Wallis test (given that BRTs do not have a phylogenetic correction), which indicated a significant relationship between citation count and family (χ^2_{19} = 32.539, p = 0.03).

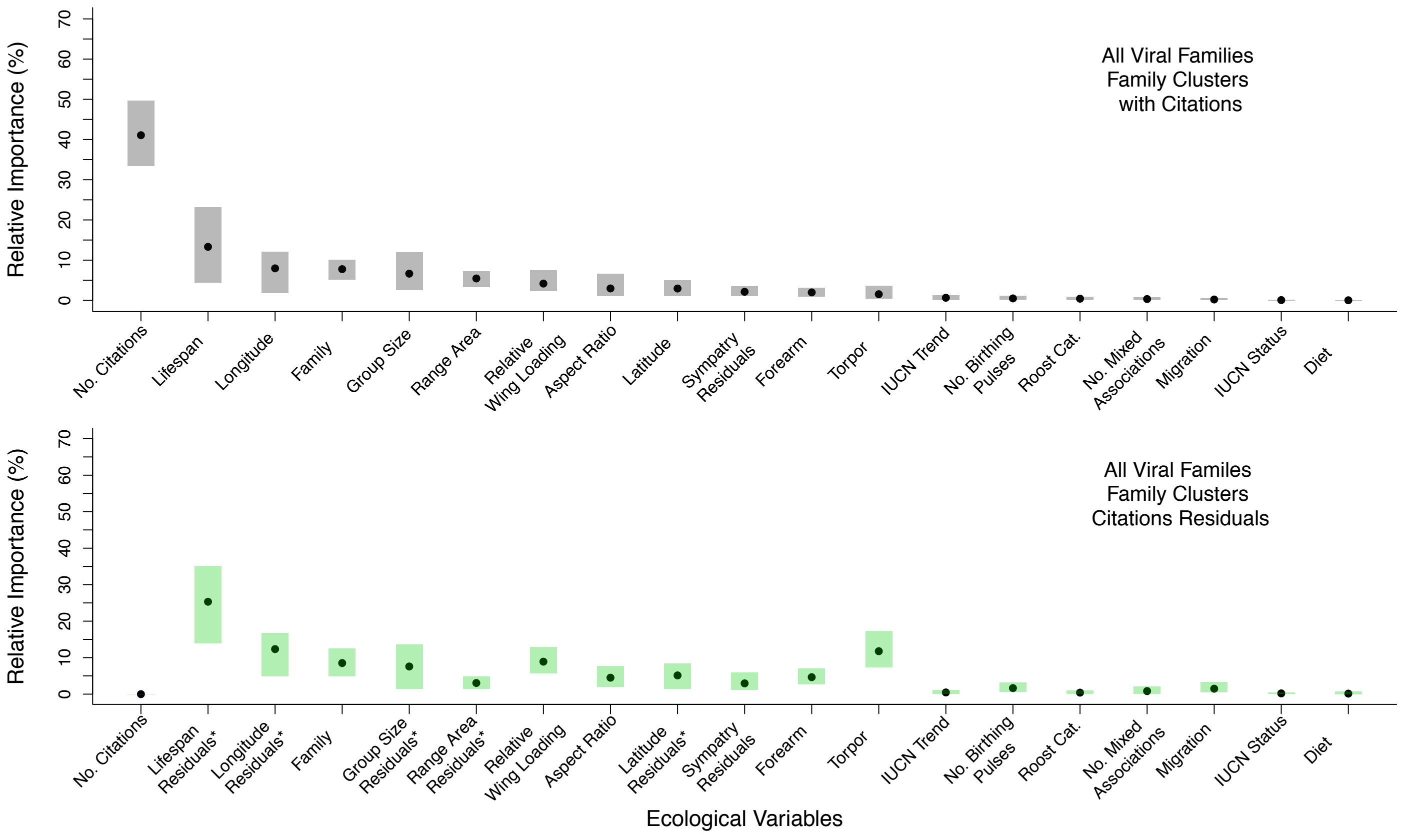

Figure A8. Relative importance of ecological traits from 200 BRT model runs. Points represent average relative importance across runs, while shaded bars represent the range within which 95% of values fall. ***Top Panel:*** Relative importance measures for predicting total viral family diversity with all ecological predictors. ***Middle Panel:*** Relative importance measures for predicting total viral family diversity without citations and including the residuals from regressions of citation count against certain ecological predictors. Ecological traits regressed against citations are marked with an asterisk and include all quantitative predictors with a correlation coefficient of at least 0.2 or (in the case of latitude) a clear, nonlinear relationship with citations (see Fig. A2). Note that we could not control for the effects of citations in the categorical variables of bat family and torpor, possibly explaining why torpor increases in importance in the residuals model.

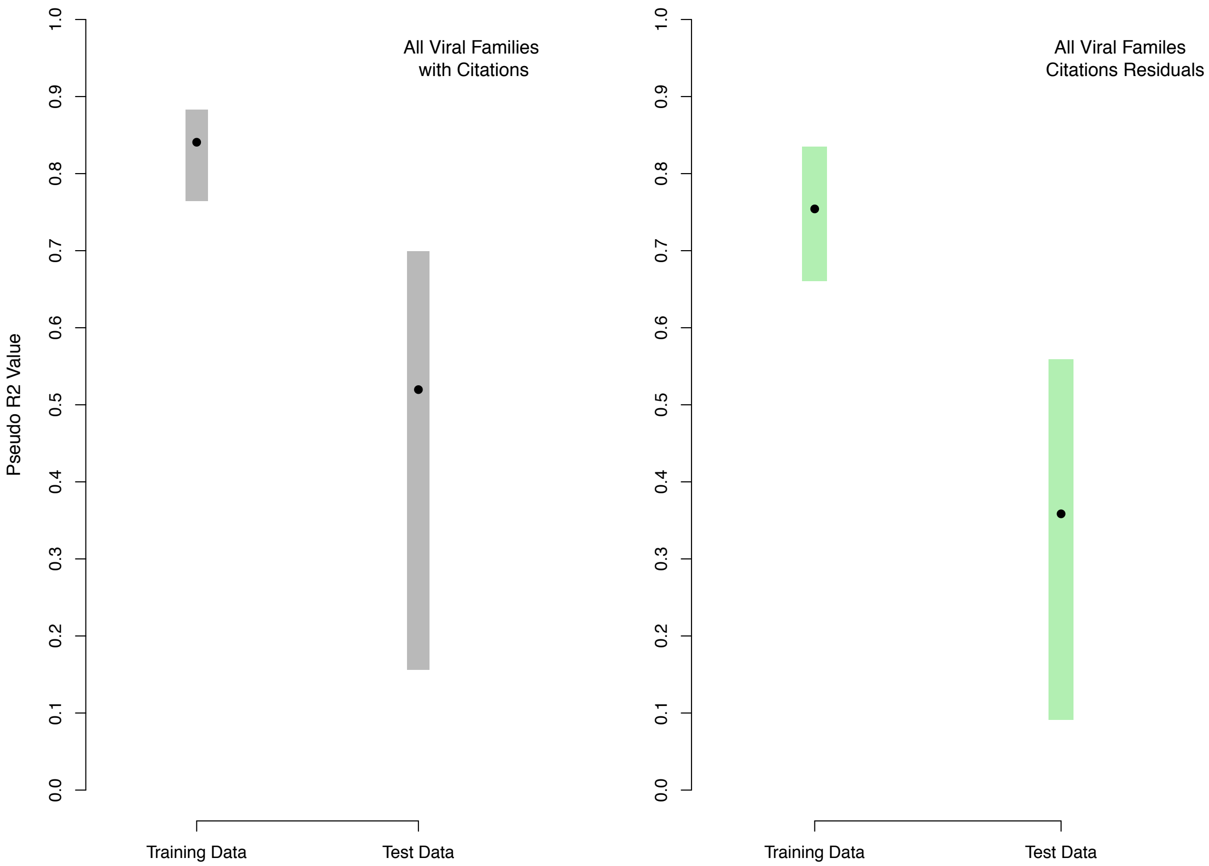

Figure A9. Measures of model performance from the 200 BRT model runs built using the full dataset. Points represent average pseudo R^2 across all runs, while shaded bars represent the range within which 95% of values fall. ***Left Panel:*** Model performance for predicting total viral family diversity with all ecological predictors. ***Right Panel:*** Model performance for predicting total viral family diversity without citations and including the residuals from regressions of citation count against certain ecological predictors. Predictive performance of the residual model, for both the training and test data, decreases only marginally relative to the model including citation count.

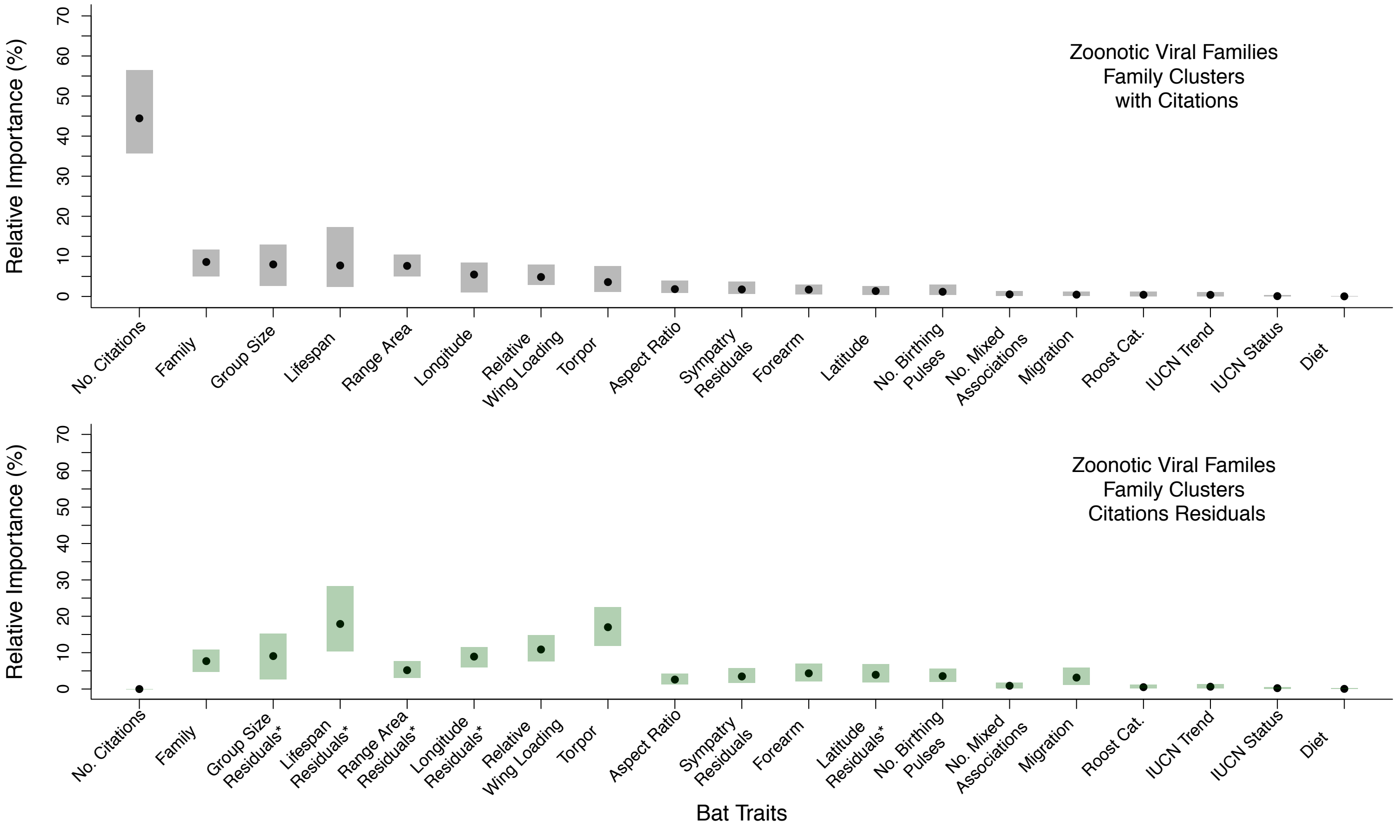

Figure A10. Relative importance of ecological traits from 200 BRT model runs. Points represent average relative importance across runs, while shaded bars represent the range within which 95% of values fall. ***Top Panel:*** Relative importance measures for predicting zoonotic viral family diversity with all ecological predictors. ***Middle Panel:*** Relative importance measures for predicting zoonotic viral family diversity without citations and including the residuals from regressions of citation count against certain ecological predictors. Ecological traits regressed against citations are marked with an asterisk and include all quantitative predictors with a correlation coefficient of at least 0.2 or (in the case of latitude) a clear, nonlinear relationship with citations (see Fig. A2). Note that we could not control for the effects of citations in the categorical variables of bat family and torpor, possibly explaining why torpor increases in importance in the residuals model.

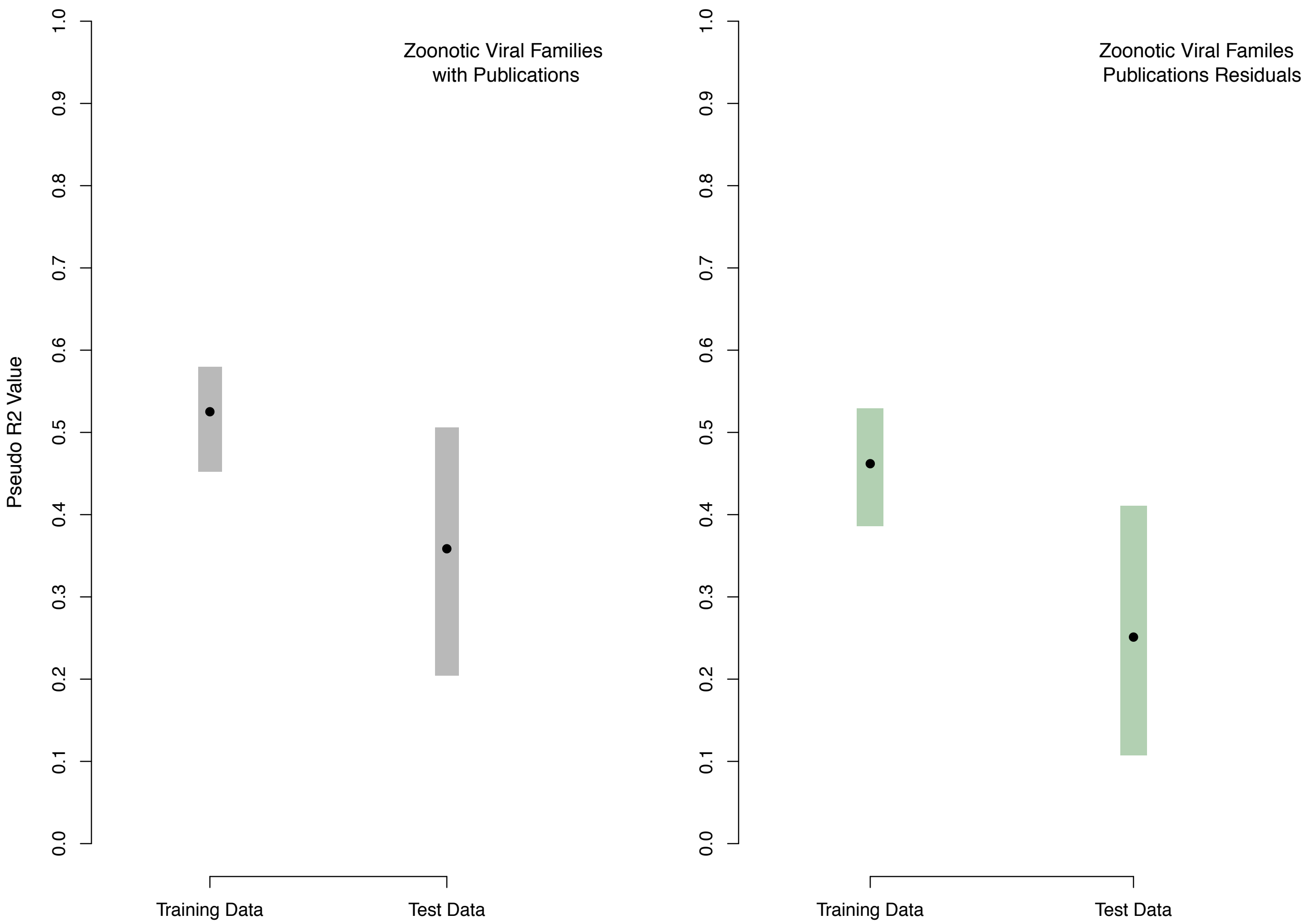

Figure A11. Measures of model performance from the 200 BRT model runs built using the full dataset. Points represent average pseudo R^2 across all runs, while shaded bars represent the range within which 95% of values fall. ***Left Panel:*** Model performance for predicting zoonotic viral family diversity with all ecological predictors. ***Right Panel:*** Model performance for predicting zoonotic viral family diversity without citations and including the residuals from regressions of citation count against certain ecological predictors. Predictive performance of the residual model, for both the training and test data, decreases slightly relative to the model including citation count. Both zoonotic viral family models have reduced predictive performance compared to the model for total viral family diversity (Fig. A9).

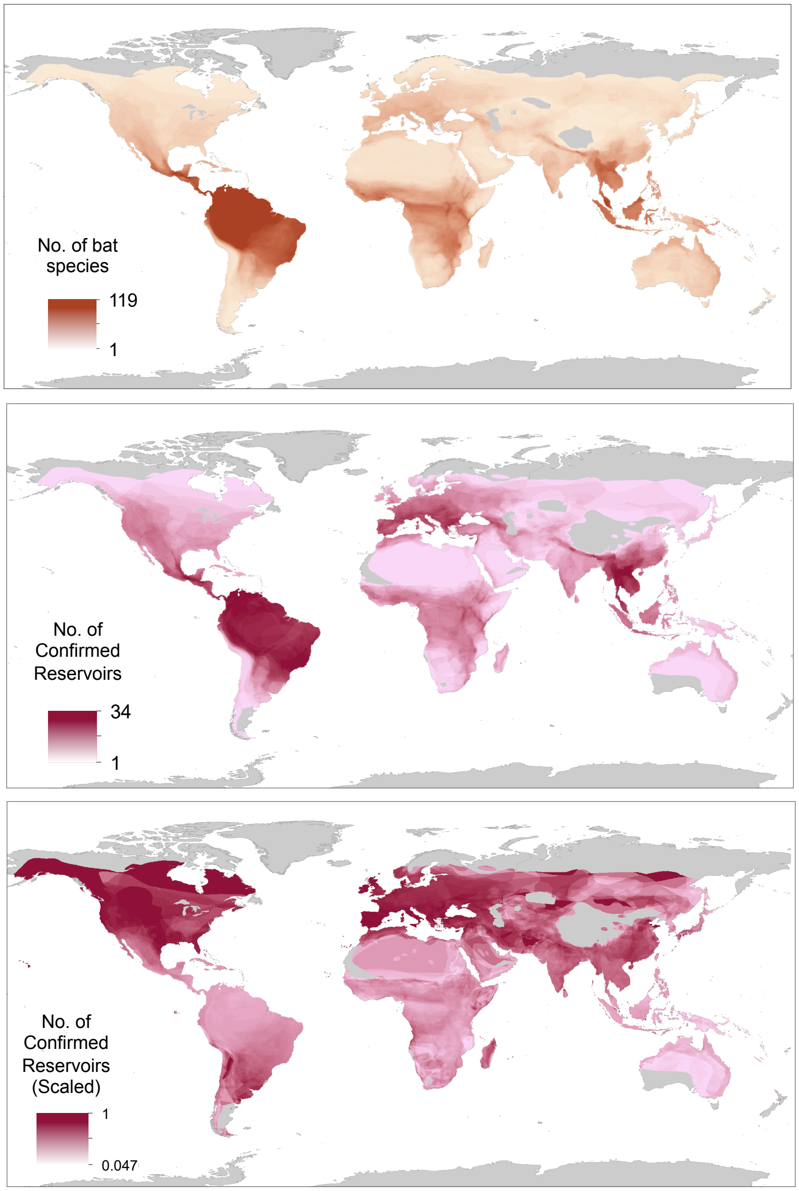

Figure A12. Global distribution of bats and confirmed viral carriers. ***Top Panel:*** Overlay map of all bat species' ranges from the IUCN. Darker regions represent areas with more bat species. ***Middle Panel:*** Overlay map of bat species known to be viral carriers. ***Bottom Panel:*** Same as middle panel, but scaled to the total bat diversity in a given area, *i.e.*, darker regions represent areas where a greater proportion of the local bat species are confirmed viral carriers.

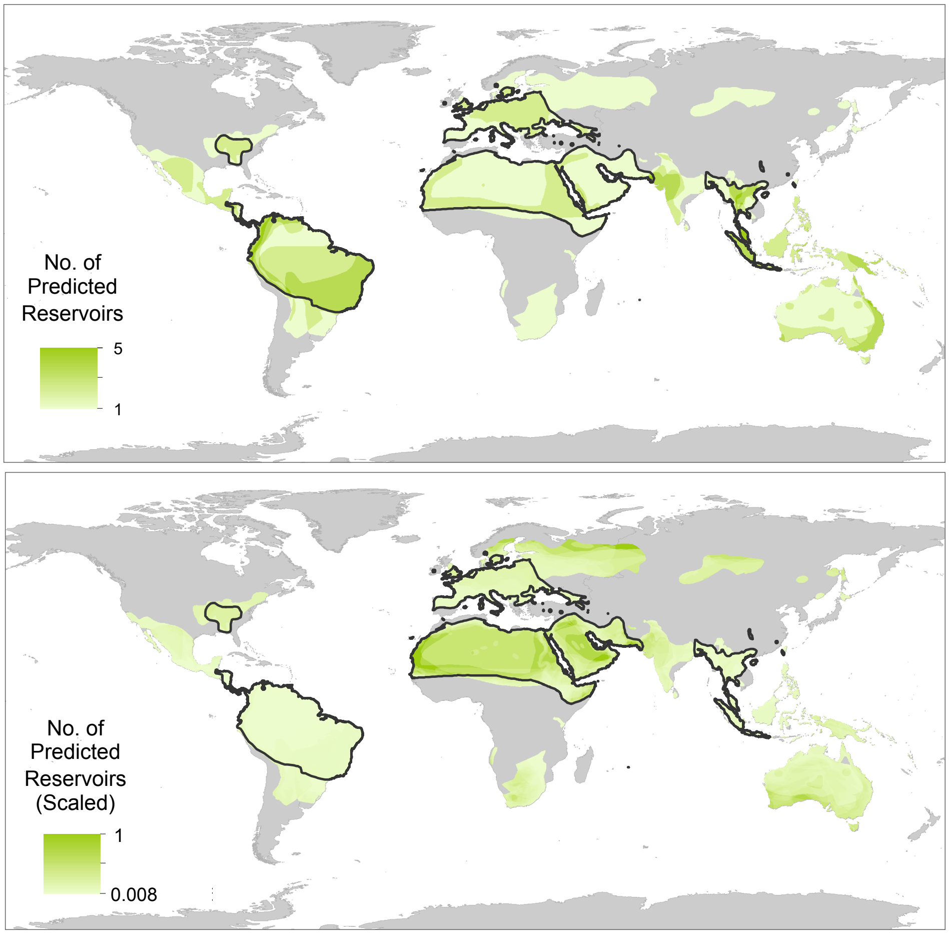

Figure A13. Global distribution of predicted viral carriers in the 95th percentile. ***Top Panel:*** Overlay map of bat species predicted to be undetected viral carriers (top 95th percentile of model predictions, averaged over 200 runs, for number of viral families hosted by bat species that are considered non-carriers in the dataset). ***Bottom Panel:*** Same as top panel, but scaled to the total bat diversity in a given area, *i.e.*, darker regions represent areas where a greater proportion of the local bat species are predicted to be undetected viral carriers. Black outlines highlight the ranges of the six bat species within the 99th percentile of model predictions. All predictions were averages of 200 BRT models.

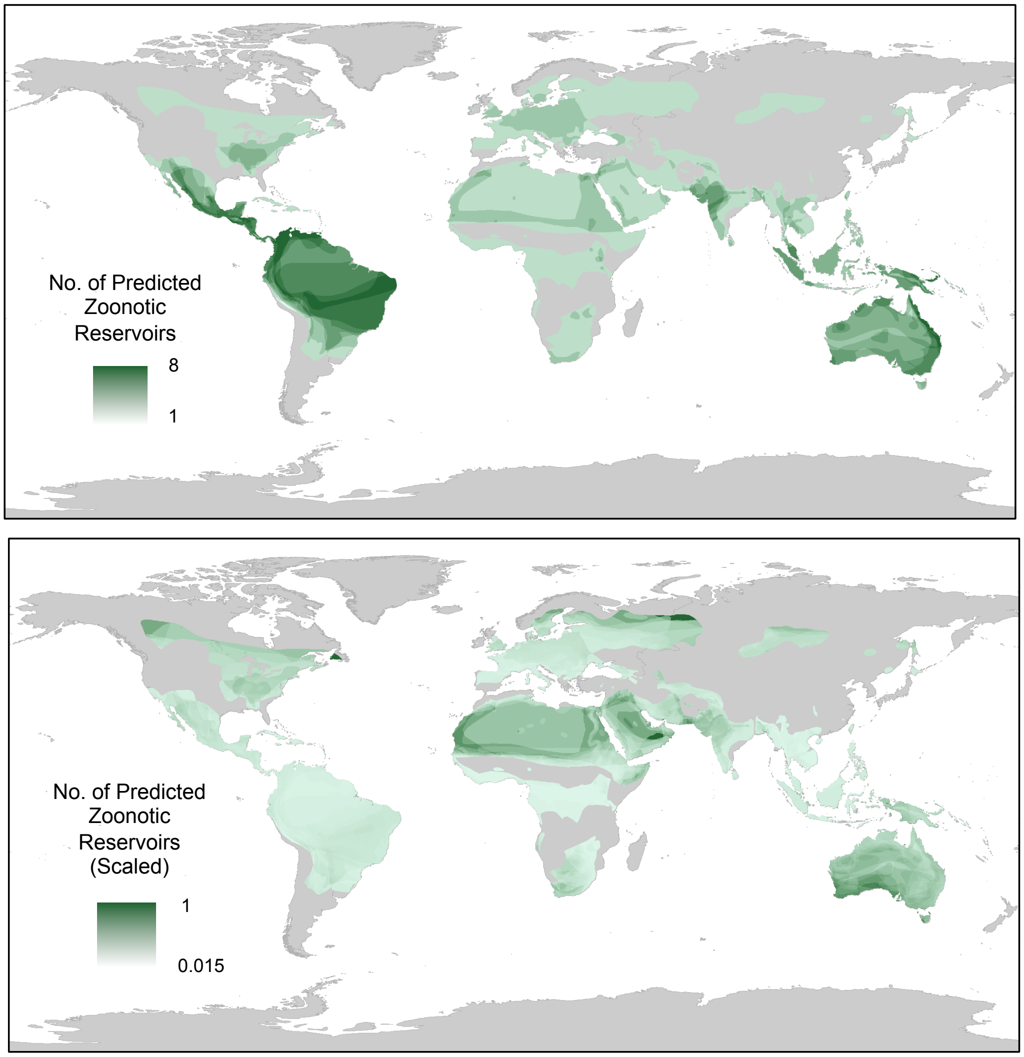

Figure A14. Global distribution of predicted zoonotic viral carriers in the 90th percentile. ***Top Panel:*** Overlay map of bat species predicted to be undetected viral carriers (top 90th percentile of model predictions, averaged over 200 runs, for number of viral families hosted by bat species that are considered non-carriers in the dataset). ***Bottom Panel:*** Same as top panel, but scaled to the total bat diversity in a given area, *i.e.*, darker regions represent areas where a greater proportion of the local bat species are predicted to be undetected viral carriers. All predictions were averages of 200 BRT models.

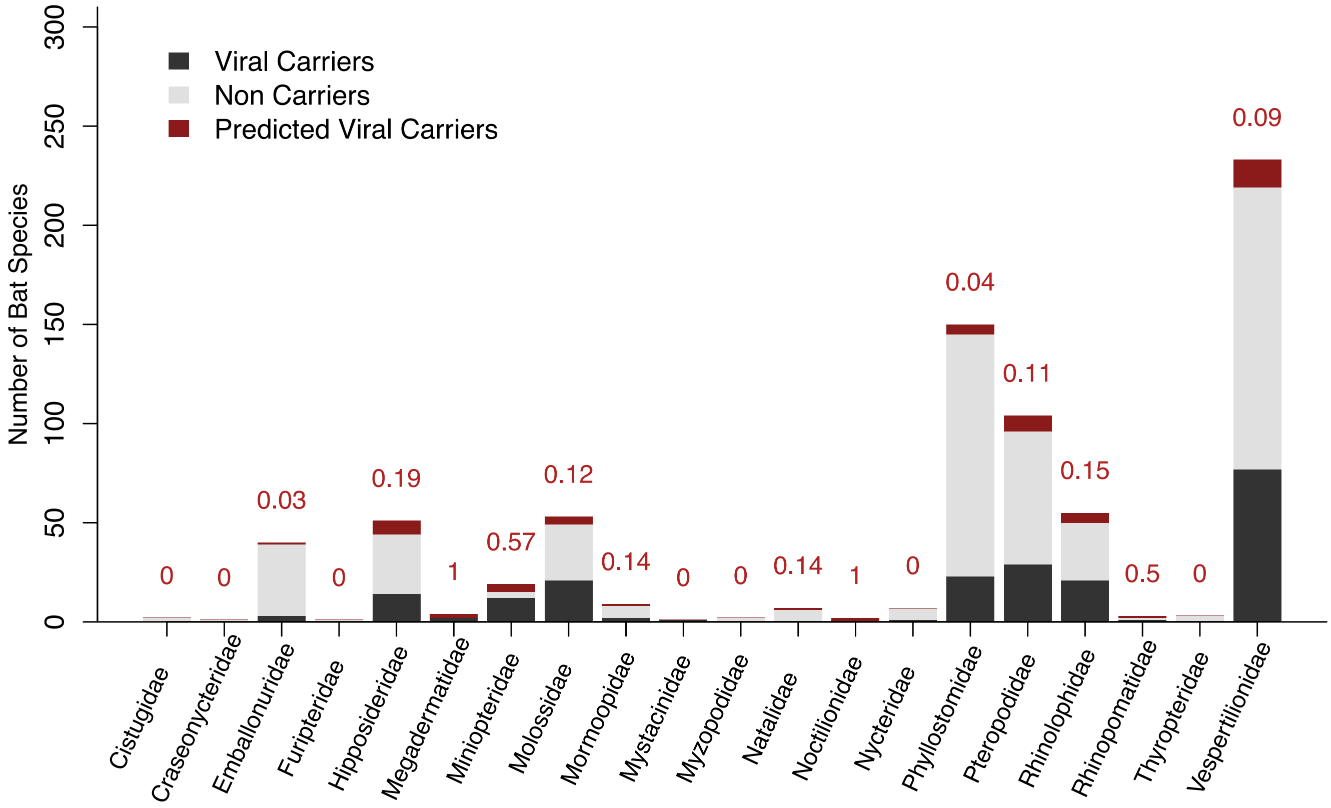

Figure A15. Numbers of species in each bat family that are either non-carriers (light gray) confirmed viral carriers (dark gray) or predicted viral carriers by our total viral family diversity BRT model (red). Height of the bar indicates the total number of species of each family included in the species' trait dataset. The proportion of non-carriers in a family predicted to be undetected viral carriers is displayed on the top of each bar in red.

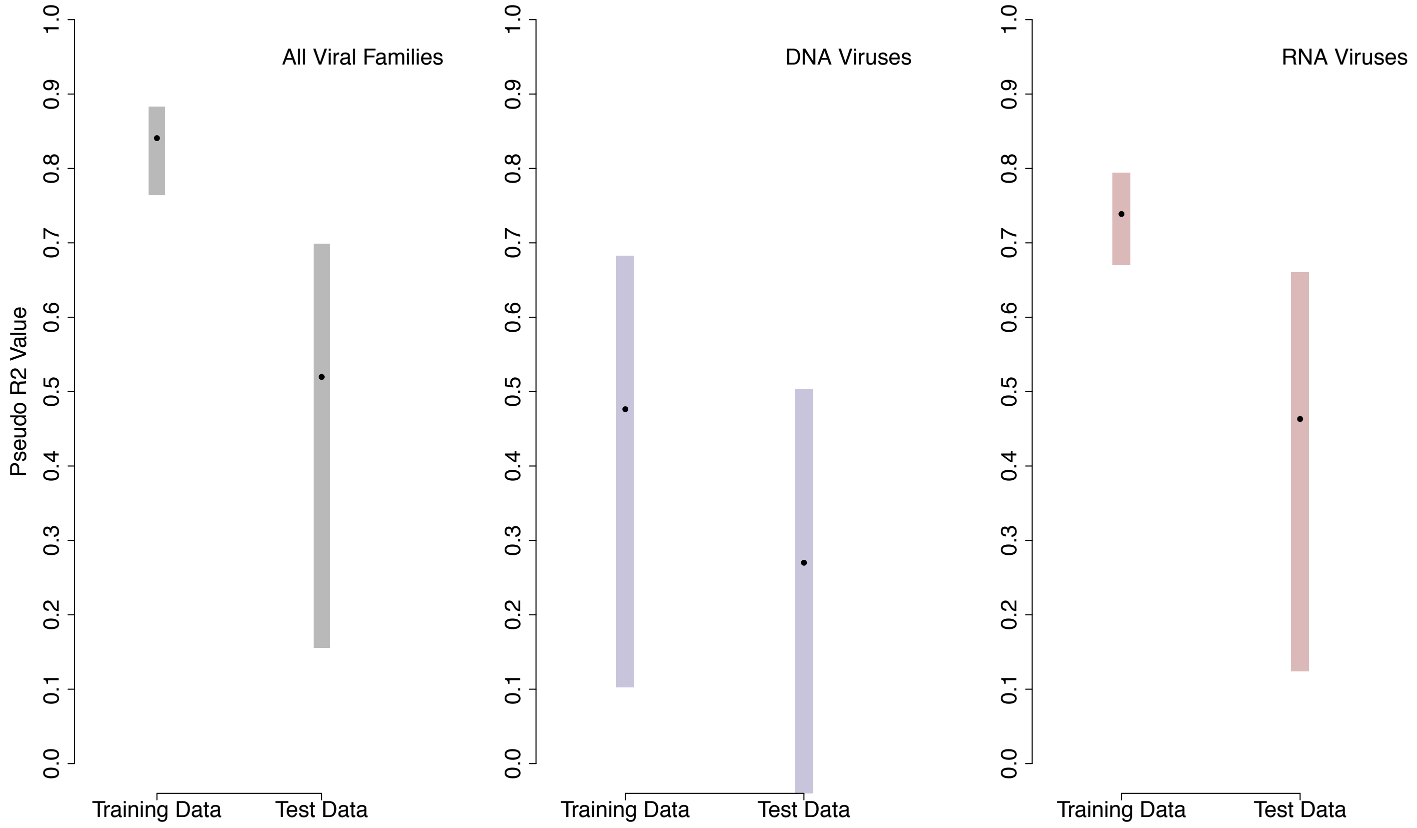

Figure A16. Measures of model performance from the 200 BRT model runs built using the full dataset (n=747 species). Points represent average pseudo R^2 across all runs, while shaded bars represent the range within which 95% of values fall. ***Left Panel:*** Model performance for predicting total viral family diversity. ***Middle Panel:*** Model performance for predicting DNA viral family diversity. ***Right Panel:*** Model performance for predicting RNA viral family diversity.

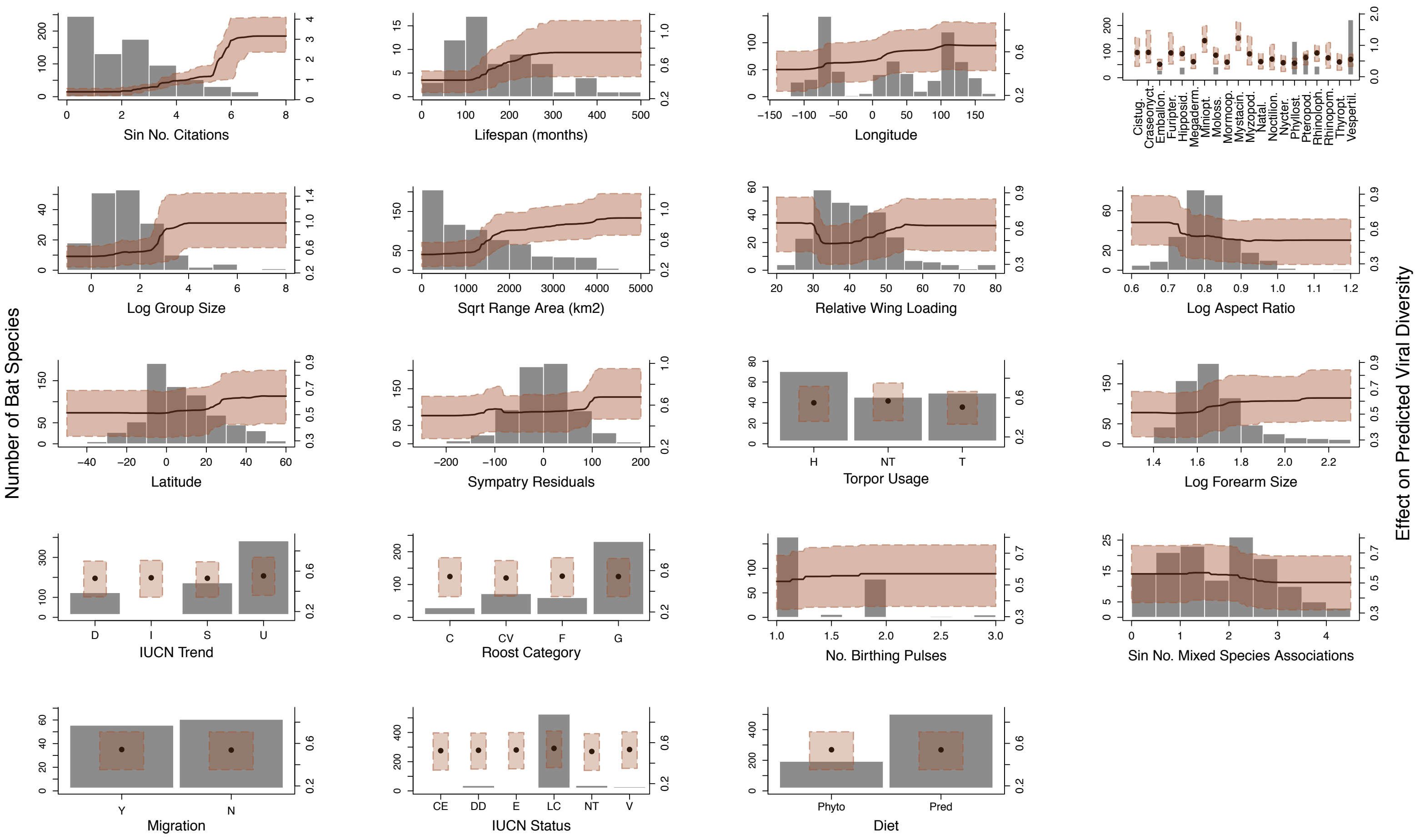

Figure A17. Partial dependence plots for all ecological traits included in the BRT models for total viral family diversity. Black lines / points show the average effect of traits on predicted viral diversity from 200 model runs, while coloured, shaded regions represent the range within which 95% of values fall. Histograms and barplots (gray) show the distribution of values for each ecological trait.

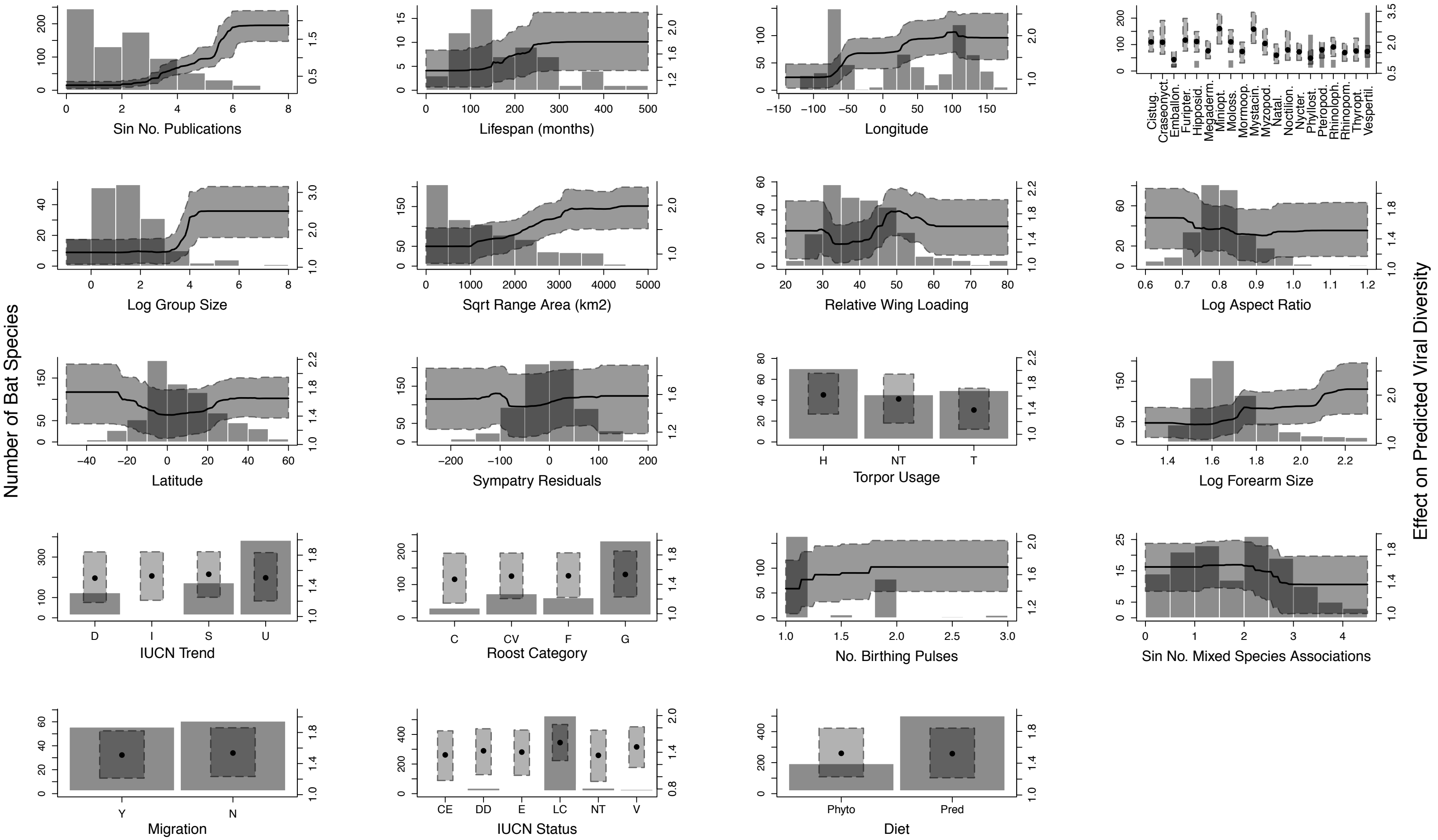

Figure A18. Partial dependence plots for all ecological traits included in the BRT models for zoonotic viral family diversity. Black lines / points show the average effect of traits on predicted viral diversity from 200 model runs, while shaded regions represent the range within which 95% of values fall. Histograms and barplots (gray) show the distribution of values for each ecological trait.

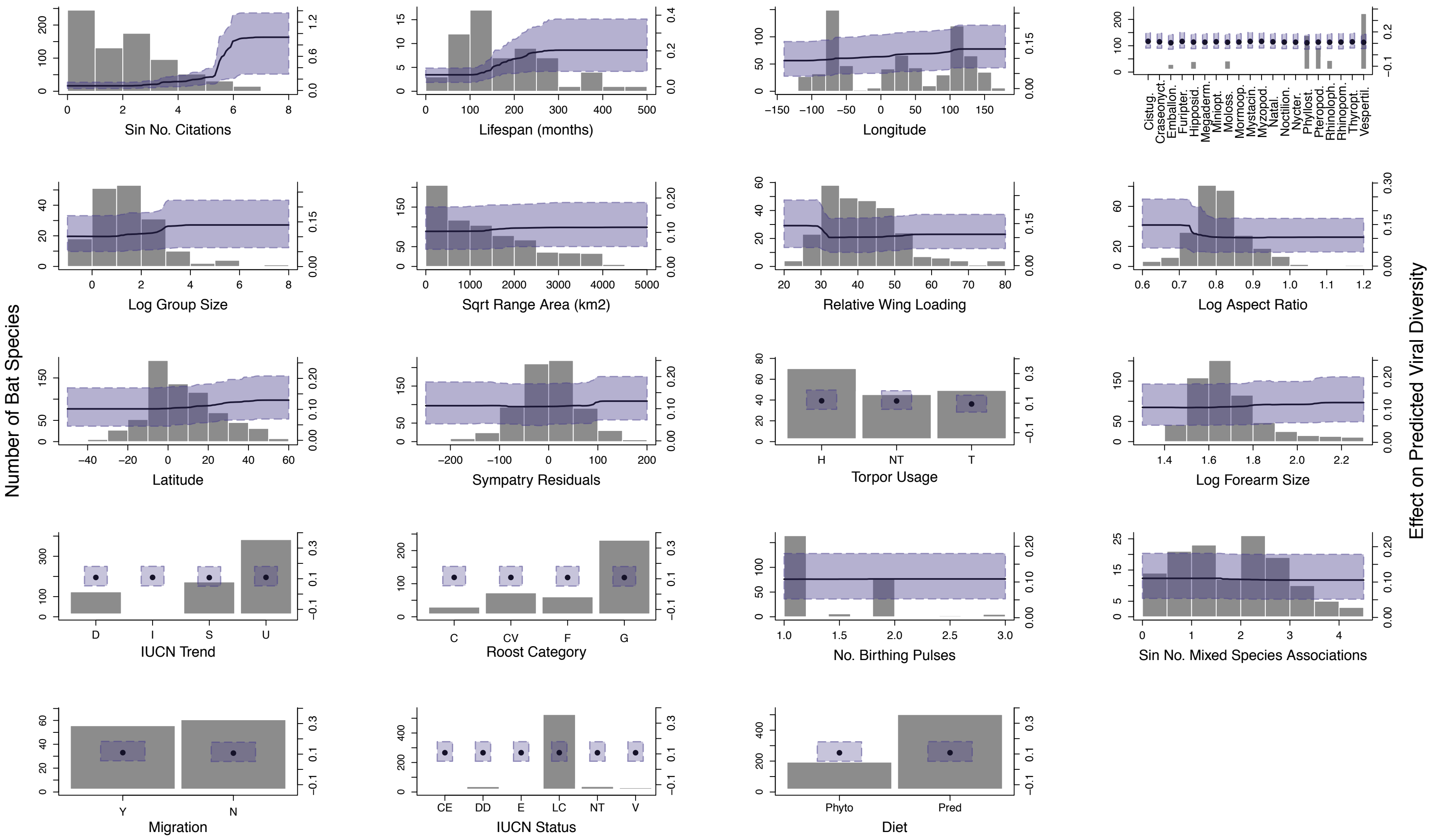

Figure A19. Partial dependence plots for all ecological traits included in the BRT model runs for DNA virus diversity. Black lines / points show the average effect of traits on predicted viral diversity from 200 model runs, while coloured, shaded regions represent the range within which 95% of values fall. Histograms and barplots (gray) show the distribution of values for each ecological trait.

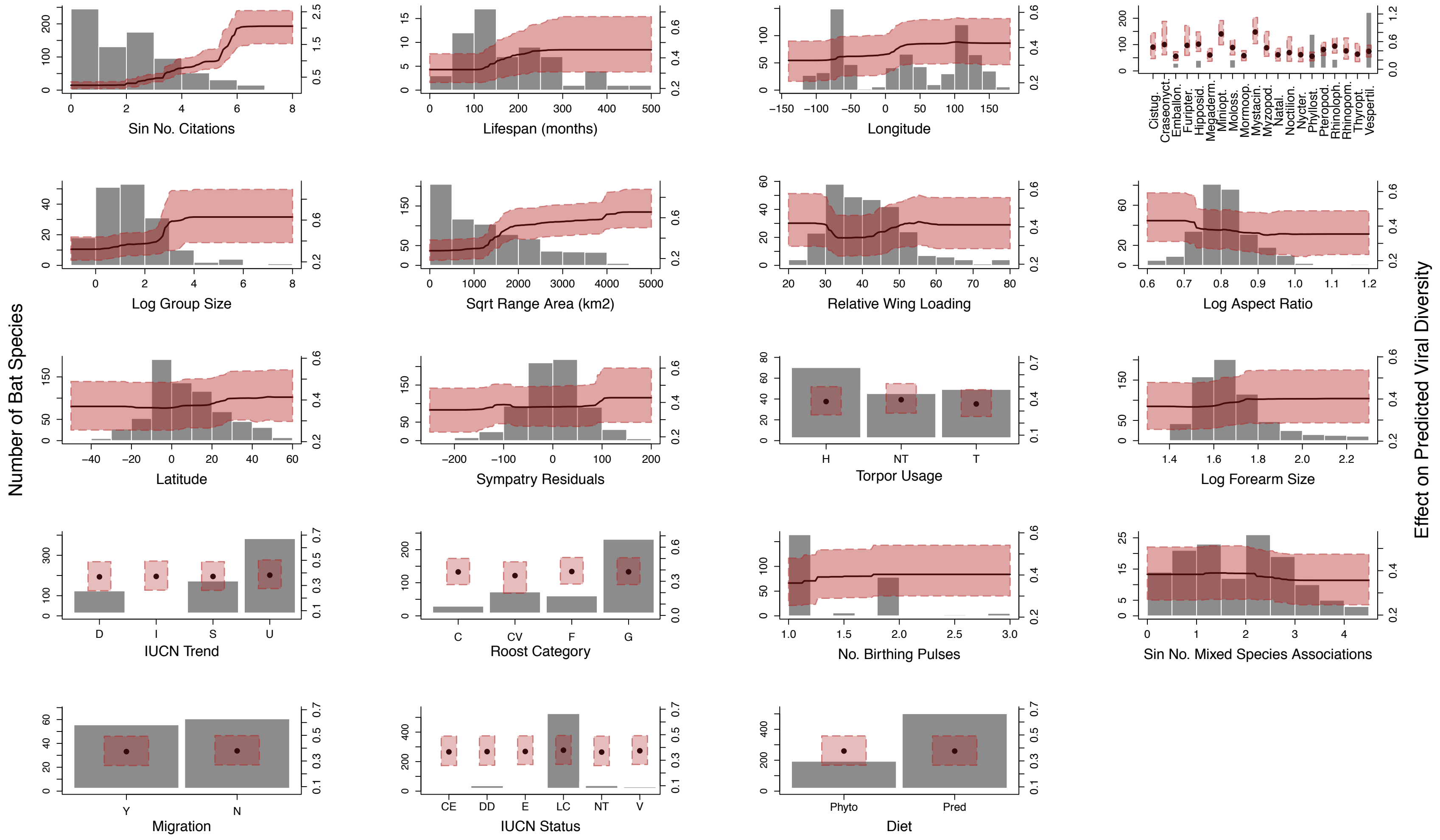

Figure A20. Partial dependence plots for all ecological traits included in the BRT models for RNA virus diversity. Black lines / points show the average effect of traits on predicted viral diversity from 200 model runs, while coloured, shaded regions represent the range within which 95% of values fall. Histograms and barplots (gray) show the distribution of values for each ecological trait.

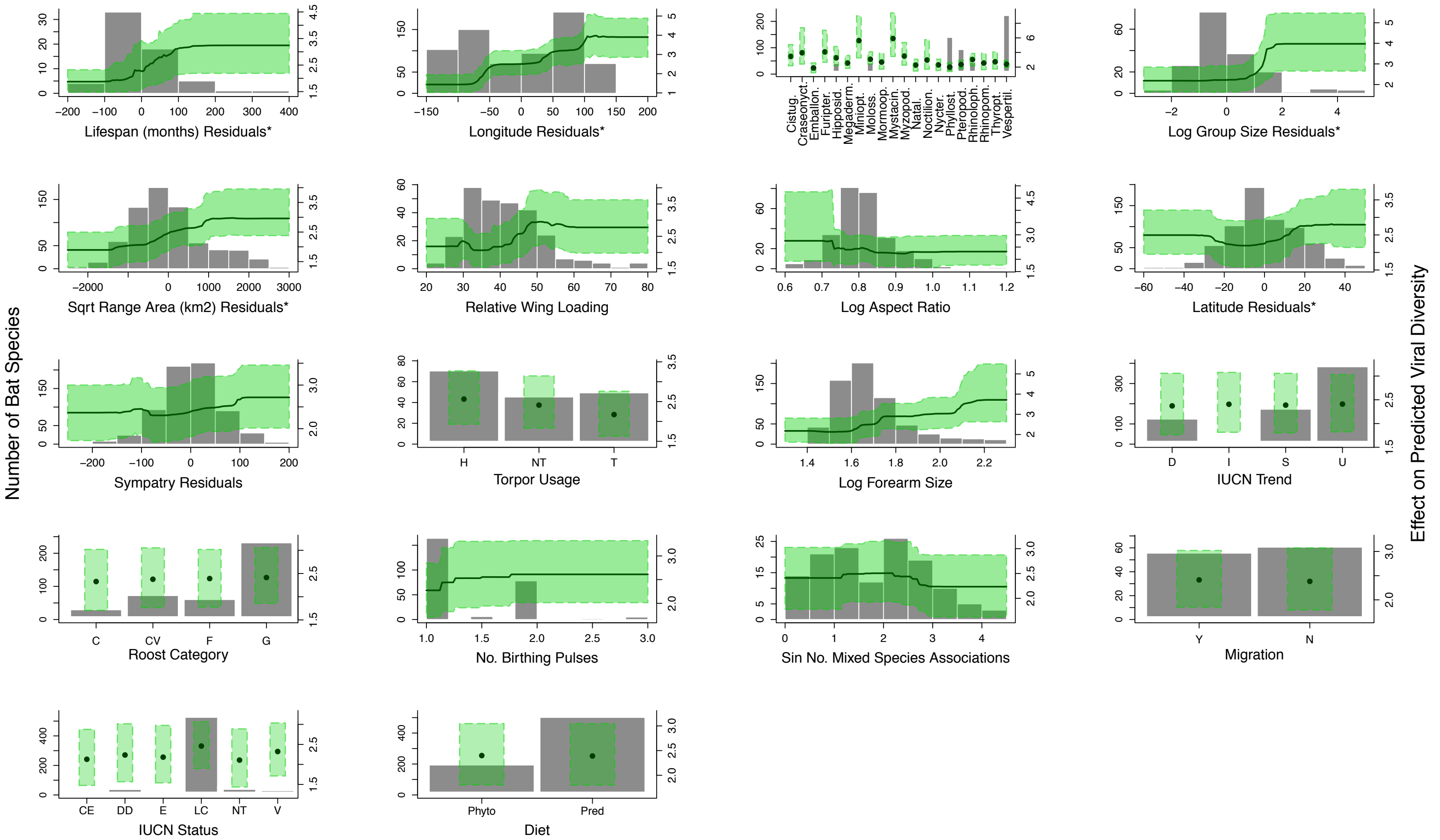

Figure A21. Partial dependence plots for all ecological traits included in the BRT models for total viral family diversity, removing the influence of citations (*i.e.*, without citation as an explicit predictor and using the residuals from regressions of citation count against certain ecological predictors – marked with an *). Black lines / points show the average effect of traits on predicted viral diversity from 200 BRT model runs, while coloured, shaded regions represent the range within which 95% of values fall within. Histograms and barplots (gray) show the distribution of values for each ecological trait.

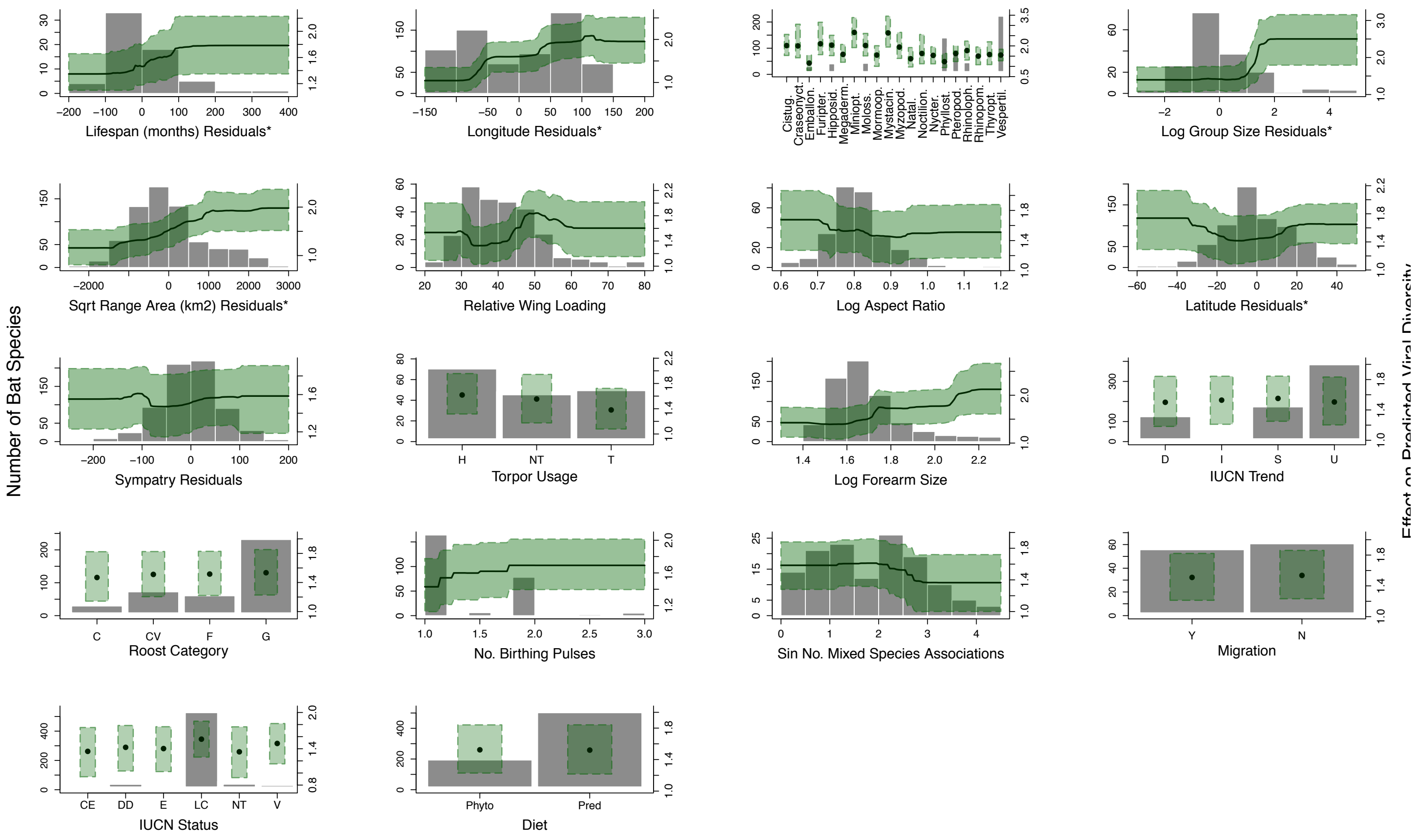

Figure A22. Partial dependence plots for all ecological traits included in the BRT models for zoonotic viral family diversity, removing the influence of citations (*i.e.*, without citation as an explicit predictor and using the residuals from regressions of citation count against certain ecological predictors – marked with an *). Black lines / points show the average effect of traits on predicted viral diversity from 200 BRT model runs, while coloured, shaded regions represent the range within which 95% of values fall within. Histograms and barplots (gray) show the distribution of values for each ecological trait.

Works Cited:

Blatteis, C. M. 2003. Fever: pathological or physiological, injurious or beneficial? Journal of Thermal Biology 28:1–13.

Brook, C. E., and A. P. Dobson. 2015. Bats as "special" reservoirs for emerging zoonotic pathogens. Trends in Microbiology 23:172–180.

Calisher, C. H., J. E. Childs, H. E. Field, K. V. Holmes, and T. Schountz. 2006. Bats: important reservoir hosts of emerging viruses. Clinical Microbiology Reviews 19:531–545.

Dobson, A. P. 2005. VIROLOGY: What Links Bats to Emerging Infectious Diseases? Science 310:628–629.

Guy, C., J. Thiagavel, N. Mideo, and J. M. Ratcliffe. 2019. Phylogeny matters : revisiting 'a comparison of bats and rodents as reservoirs of zoonotic viruses.' Royal Society Open Science 6:181182.

Hasday, J. D., K. D. Fairchild, and C. Shanholtz. 2000. The role of fever in the infected host. Microbes and Infection 2:1891–1904.

Hayman, D. T. S. 2015. Biannual birth pulses allow filoviruses to persist in bat populations. Proceedings of the Royal Society B: Biological Sciences 282:20142591.

Kamiya, T., K. O'Dwyer, S. Nakagawa, and R. Poulin. 2014. What determines species richness of parasitic organisms? A meta-analysis across animal, plant and fungal hosts. Biological Reviews 89:123–134.

Lindenfors, P., C. L. Nunn, K. E. Jones, A. A. Cunningham, W. Sechrest, and J. L. Gittleman. 2007. Parasite species richness in carnivores: effects of host body mass, latitude, geographical range and population density. Global Ecology and Biogeography 16:496–509.

Luis, A. D., D. T. S. Hayman, T. J. O'Shea, P. M. Cryan, A. T. Gilbert, J. R. C. Pulliam, J. N.

Mills, M. E. Timonin, C. K. R. Willis, A. A. Cunningham, A. R. Fooks, C. E. Rupprecht, J. L. N. Wood, and C. T. Webb. 2013. A comparison of bats and rodents as reservoirs of zoonotic viruses: are bats special? Proceedings of the Royal Society B: Biological Sciences 280:20122753.

Luis, A. D., T. J. O'Shea, D. T. S. Hayman, J. L. N. Wood, A. A. Cunningham, A. T. Gilbert, J. N. Mills, and C. T. Webb. 2015. Network analysis of host-virus communities in bats and rodents reveals determinants of cross-species transmission. Ecology Letters 18:1153–1162.

Norberg, U. M., and J. V. M. Rayner. 1987. Ecological morphology and flight in bats (Mammalia: Chiroptera): wing adaptations, flight performance, foraging strategy, and echolocation. Philosophical Transactions of the Royal Society B 316:335–427.

O'Shea, T. J., P. M. Cryan, A. A. Cunningham, A. R. Fooks, D. T. S. Hayman, A. D. Luis, A. J. Peel, R. K. Plowright, and J. L. N. Wood. 2014. Bat flight and zoonotic viruses. Emerging Infectious Diseases 20:741–745.

Olival, K. J., P. R. Hosseini, C. Zambrana-Torrelio, N. Ross, T. L. Bogich, and P. Daszak. 2017. Host and viral traits predict zoonotic spillover from mammals. Nature 546:646–650.

Poulin, R., and S. Morand. 2004. Parasite biodiversity. Smithsonian Books, Washington, DC.

Revell, L. J. 2012. phytools: an R package for phylogenetic comparative biology (and other things). Methods in Ecology and Evolution 3:217–223.

Shi, J. J., and D. L. Rabosky. 2015. Speciation dynamics during the global radiation of extant bats. Evolution 69:1528–1545.

Sooryanarain, H., and S. Elankumaran. 2015. Environmental Role in Influenza Virus Outbreaks. Annual Review of Animal Biosciences 3:347–373.

Turmelle, A. S., and K. J. Olival. 2009. Correlates of viral richness in bats (Order Chiroptera).

EcoHealth 6:522–539.

Webber, Q. M. R., Q. E. Fletcher, and C. K. R. Willis. 2017. Viral richness is positively related to group size, but not mating system, in bats. EcoHealth 14:652–661.

Willig, M. R., D. M. Kaufman, and R. D. Stevens. 2003. Latitudinal Gradients of Biodiversity: Pattern, Process, Scale, and Synthesis. Annual Review of Ecology, Evolution, and Systematics 34:273–309.
