# Supplementary material for: The influence of bat ecology on viral diversity and reservoir status
Source: Ecol Evol. 2020 May 8;10(12):5748–58. doi: 10.1002/ece3.6315 (PMC7319232; doi:10.1002/ece3.6315)
Supplement: Supplementary file 2 — Appendix B [file ECE3-10-5748-s002.pdf]

B.1 Effect of Species' Data Coverage on Boosted Regression Tree Models

Boosted regression trees (BRTs) are advantageous for comparative studies as they can accommodate missing data (Elith et al. 2008). Although we were able to collect some trait information for all 812 bat species in the Shi and Rabosky (2015) phylogeny, there was substantial variance in the data available for different species (*e.g.*, we have information on all 19 predictors for some well-studied species, but only two or three predictors for others). We therefore examined how including species for which we had differing amounts of trait information affected the relative importance and ordering of ecological predictors, as well as the predictive performance of BRT models.

Varying Species' Trait Data Coverage: From the full species' trait dataset containing all 812 species, we created three sub-datasets with varying numbers of species. The first contained only bat species for which we had information on five or more ecological traits (at least 25% of traits included in the full analysis). This resulted in a dataset containing ~90% of species in the Shi & Rabosky (2015) phylogeny (n=747 bat species). The second dataset contained bat species for which we had information on 10 or more ecological traits (≥ 50% of all traits), resulting in inclusion of ~75% of the 812 species (n=615 bat species). The final dataset contained species for which we had information on 12 or more ecological traits (≥60% of all traits), resulting in inclusion of ~50% of the 812 species (n =357 bat species). We partitioned each of these sub-datasets into training (80% of species) and test (20% of species) sets and built a BRT model using all ecological predictors, family as our phylogenetic grouping, and number of viral families as the response. For each of the three sub-datasets, we repeated this procedure 200 times (with different training and test partitions), recording measures of variable relative importance and model performance for each run. Analyses were completed in R (*v. 3.4.4*; R Core

Development Team, 2014). As outlined in the main text, we optimized shrinkage, learning rate, and number of trees for each model using the *caret* package (*v. 6.0-79*; Kuhn et al., 2018) and built final BRT models using the *gbm* package (*v. 2.0-8*; Ridgeway, 2017).

Results

Overall, including species in the analysis for which we had different amounts of trait data did not substantially alter the ordering of ecological predictors in BRT models (Fig. B1). Although there were small shifts in relative importance, in all cases, the top 10 predictors remained the same (Fig. B1). Average metrics of model performance were also similar when comparing BRT models built using the three different sub-datasets (Fig. B2), though predictive performance on the test data was lower on average and more variable when fewer of the species were included (*i.e.*, 50% of the Shi and Rabosky (2015) species, each with data for at least 12 ecological traits; Fig. B2).

Conclusions

Given that the relative importance and ordering of ecological predictor variables does not substantially shift in BRT models built using species for which there are varying amounts of trait data, but predictive power declines with fewer species, in the main text we present the analyses that include the most species (*i.e.*, ~90% of the Shi and Rabosky (2015) species), despite the limited trait coverage (*i.e.*, at least ~25% of the ecological traits are known for each species).

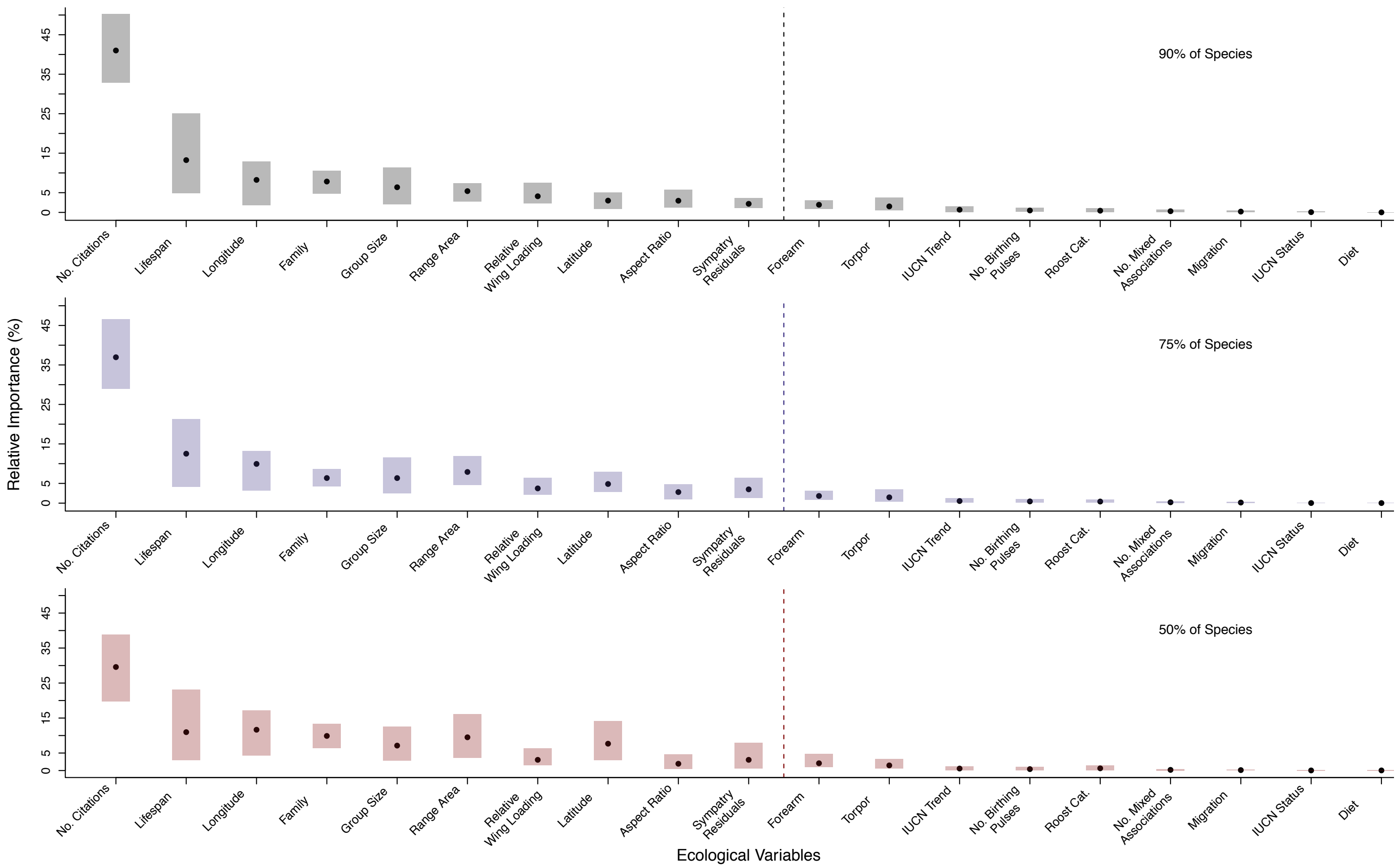

Figure B1. Relative importance of ecological traits from the 200 BRT model runs built using species for which we had differing amounts of ecological trait data (top: at least 25% of traits recorded per species; middle: at least 50% of traits; bottom: at least 60% of traits). Points represent average relative importance, while shaded bars represent the range within which 95% of values fall. Dashed lines separate predictors that contribute ~95% to the final model (calculated using mean relative importance).

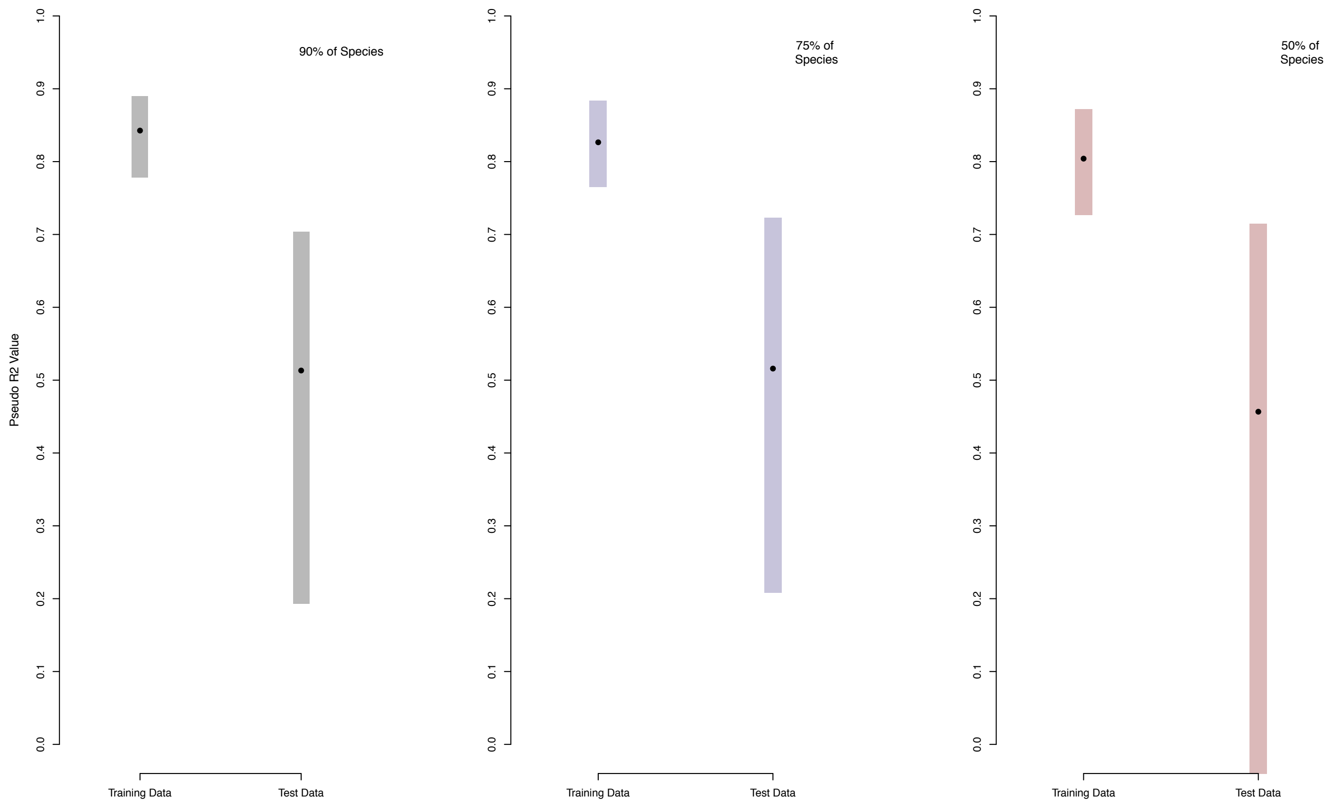

Figure B2. Measures of model performance from the 200 BRT model runs built using different percentages of the full species' dataset, for which we had differing amounts of ecological trait data (as in Fig. B1). Points represent average pseudo R^2, while shaded bars represent the range within which 95% of values fall.

B.2 Effect of Zeros on Boosted Regression Tree Models

For all boosted regression tree (BRT) models, we designated species for which we could find no viral information as zeros. This was the same method adopted by Han *et al.* (2016, 2015) who argue that designating such species as zeros, rather than NAs, is conservative and allows for the development of predictive models that can be improved upon in the future. While we agree with this perspective, we also examined how the inclusion of zeros in BRT models affected the relative importance and ordering of ecological predictors, as well as the predictive performance of models. To accomplish this, we ran several variations of our BRTs.

[1] All Zeros: We split our full dataset, containing all zeros, into training (80% of species) and test (20% of species) datasets and built a BRT model. We included all ecological predictors, family as our phylogenetic grouping, and number of viral families as the response. We repeated this procedure 200 times, recording measures of variable relative importance and model performance for each run. This is the same analysis presented in the main text and is included here for comparison.

[2] Equal Zeros: We isolated viral carriers (n=207 species) and drew an equal-sized random sample of the species for which we had no viral information (*i.e.*, 207 of a possible 540 species). We split the combined species set (414 total species) into training (80% of species) and test (20% of species) datasets and built a BRT model using all ecological predictors and number of viral families as the response. We repeated this procedure 200 times, taking a different random sample of the zeros at each iteration, to examine if the identity of the non-reservoirs had an effect on model outcomes. For each model run, we recorded measures of variable relative importance and model performance (*i.e.*, training and test pseudo R^2). We performed this equal zero sampling separately for each of the three phylogenetic grouping predictors outlined in the

main text (i.e., family, the 56MYA phylogenetic, and the 41MYA phylogenetic clusters). Given that the phylogenetic grouping did not substantially alter the ordering of ecological traits in models (Fig. B3) or predictive performance of models (Fig. B4), all additional analyses include only family as a predictor.

[3] Zeros with Citations: We isolated species from the dataset that were either known viral carriers or, if they were non-reservoirs (*i.e.,* a zero), had at least one citation. Like other authors (*e.g.* Lindenfors et al., 2007; Luis et al., 2013; Nunn et al., 2014; Olival et al., 2017) we include citation count as a proxy for study effort. Thus, including only those zeros that have been "studied" may be a more conservative approach than including all zeros. This partition resulted in a dataset of 627 species (207 viral carriers, 420 non-viral carriers). We split these 627 species into training (80% of species) and test (20% of species) datasets and built a BRT model. We included all ecological predictors, family as our phylogenetic grouping, and number of viral families as the response. We repeated this procedure 200 times, recording measures of variable relative importance and model performance for each run.

[4] No Zeros: In this set of analyses we considered only those species that were known viral carriers (n=207 species). We partitioned these species into training (80% of species) and test (20% of species) datasets and built a BRT model that included all ecological predictors, family as our phylogenetic grouping, and number of viral families as the response. We repeated this procedure 200 times, recording measures of variable relative importance and model performance for each run.

Results

Overall, including either no zeros ([4] above) or a sampling of zeros ([2] and [3] above) in BRTs did not substantially alter the ordering of ecological predictors in the models (Fig. B3 &

B5). In all cases, the top 10 predictors in BRT models remained the same (Fig. B5). There were small shifts in the relative importance of certain ecological traits when only species who carried viruses were considered (Fig. B5; lower panel). In this case, the ecological traits of longitude, latitude, aspect ratio, forearm size, IUCN trend, and roost category had slightly higher average relative importance compared to BRT models built using all of the zeros ([1] above). Number of citations had a lower average relative importance than in the all zero models. Despite using substantially less data to inform the BRT models, the range 95% of relative importance values fall within in the no zero models still overlap with the range that 95% of relative importance values fall within for the all zero BRT models. Measures of ecological trait relative importance were similar when comparing BRT models built using all of the zeros to a random sampling of 207 of the zeros or only those zeros that had at least one citation (see Fig. B5, top and middle panels).

As with measures of relative importance, average metrics of model performance were similar when comparing BRT models built using all of the zeros or a random sampling of 207 of the zeros (Fig. B6). The predictive performance of the model on the training dataset was more variable in BRT models that either sampled a varying number of zeros or considered no zeros, relative to the predictive performance of the BRT models built using the full dataset. As would be expected for training BRT models on smaller subsets of data, predictive performance for the test data was lower and/or more variable for analyses using only “studied zeroes” [3] or no zeros [4], compared to using all zeroes [1]. The one exception to this is that models using an equal number of zeros as non-zeros [2] had less variance in performance on test data.

Conclusions

Regardless of the number of zeros included in BRT models, the relative importance and ordering of ecological predictor variables does not substantially shift, nor affect the conclusions drawn. However, inclusion of zeros does, in general, seem to improve the predictive performance of our BRT models. Thus, like Han *et al.* (2016, 2015), we feel it is appropriate to include all of these assumed non-reservoir species in our final model building.

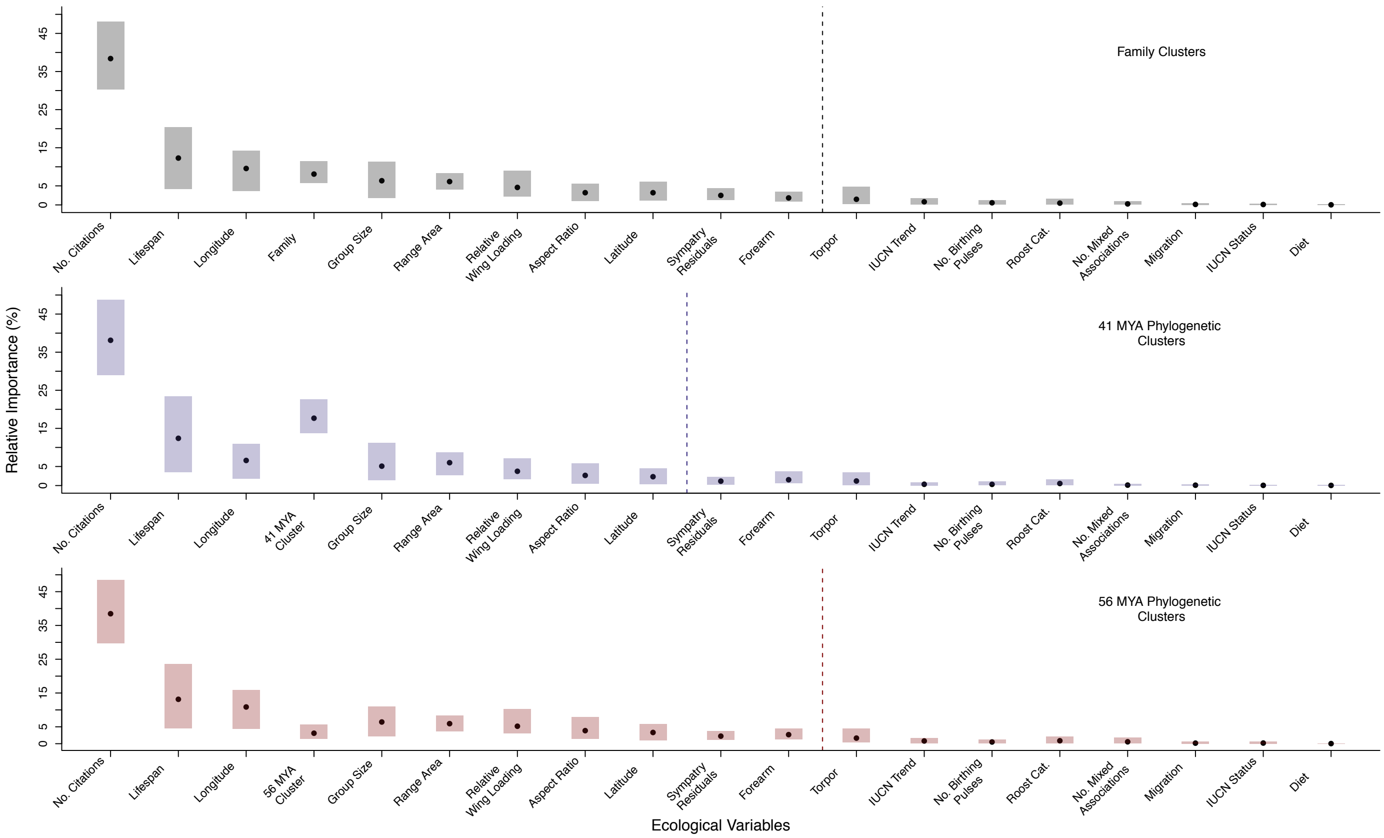

Figure B3. Relative importance of ecological traits from the 200 BRT model runs built using equal numbers of viral and non-viral carriers (*i.e.,* a random sample of 205 of the zeros in the dataset, resampled for each of the 200 models). Points represent average relative importance, while shaded bars represent the range within which 95% of values fall. This equal zero sampling procedure was performed three times, each time including a different phylogenetic grouping (*i.e.,* family, 41 MYA phylogenetic clusters, or 56 MYA phylogenetic clusters) as a predictor. Dashed lines separate predictors that contribute ~95% to the final model (calculated using mean across runs).

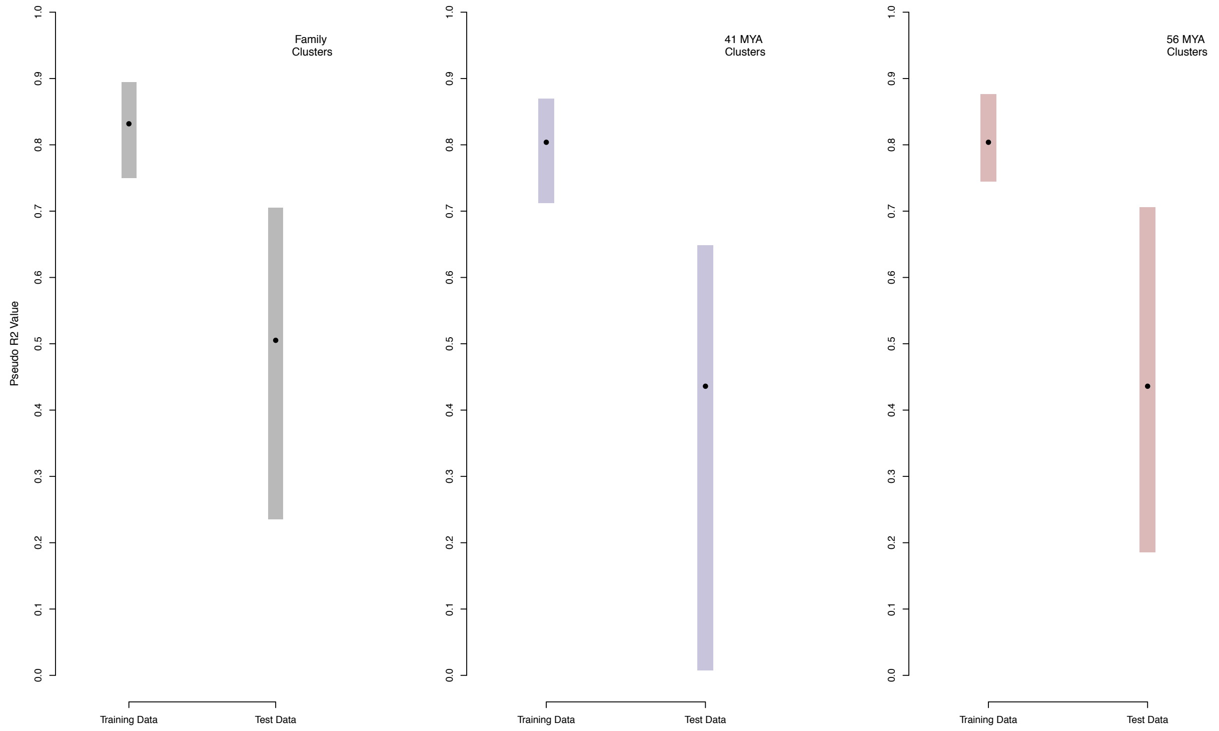

Figure B4. Measures of model performance from the 200 BRT model runs built using equal numbers of viral and non-viral carriers (*i.e.,* a random sample of 205 of the zeros in the dataset, resampled for each of the 200 models). Points represent average pseudo R^2, while shaded bars represent the range within which 95% of values fall. This equal zero sampling procedure was performed three times, each time including a different phylogenetic grouping (*i.e.,* family, 41 MYA phylogenetic clusters, or 56 MYA phylogenetic clusters) as a predictor.

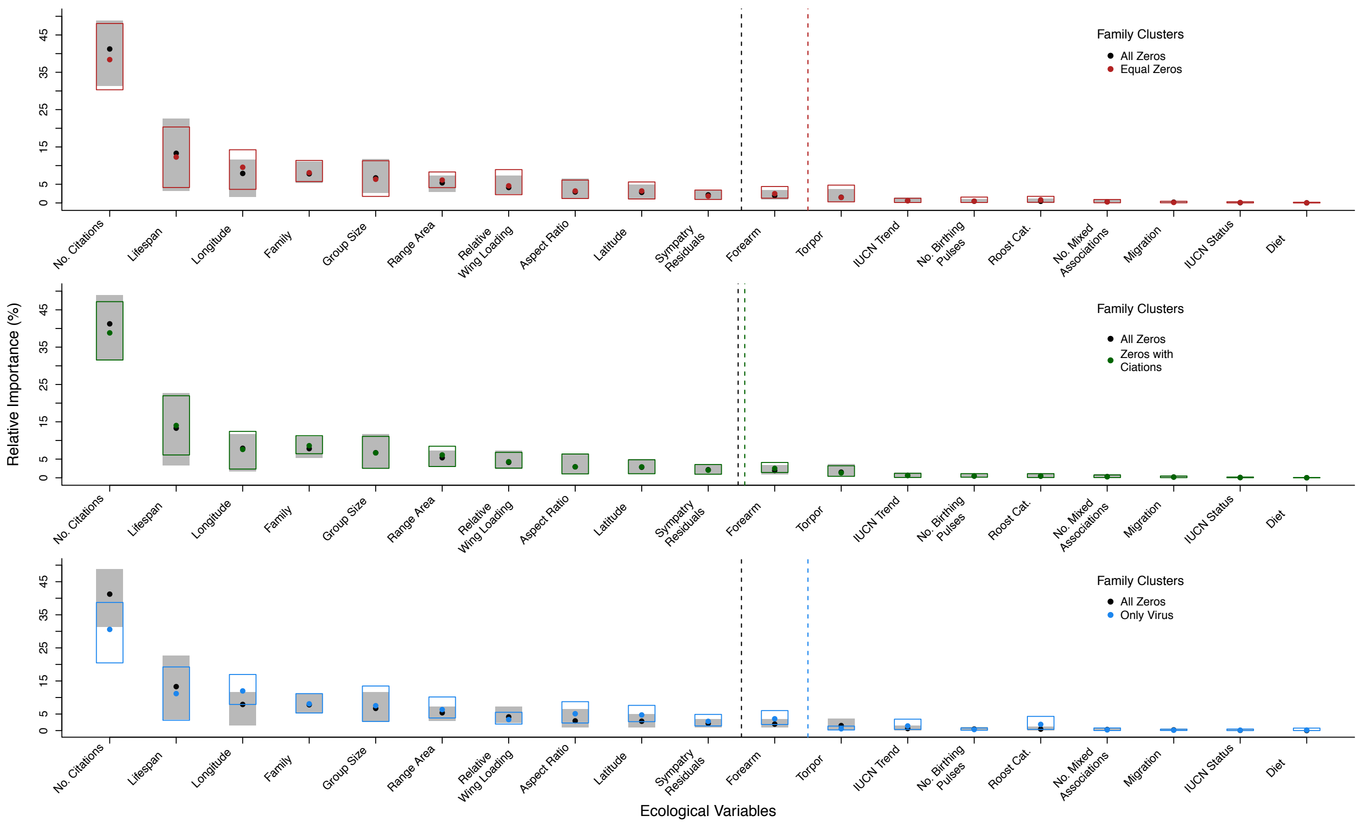

Figure B5. Measures of the relative importance of ecological variables from the BRT model runs built using the methods described in [1-4] above. Points represent average relative importance, while shaded or outlined bars represent the range within which 95% of values fall. Dashed lines separate predictors that contribute ~95% to the final model (calculated using mean across runs). In all panels, results are compared with values for BRT models build using all zeros ([2]; black points, gray regions). ***Top Panel***: Results for BRT models built using a random sampling of 205 of the zeros in the dataset ([1]; red points and boxes). ***Middle Panel:*** Results for BRT models built using only zeros that also had at least one citation ([3]; green points and boxes). ***Bottom Panel:*** Results for BRT models built using only confirmed reservoir species (*i.e.*, non-zeros, [4]; blue points and boxes).

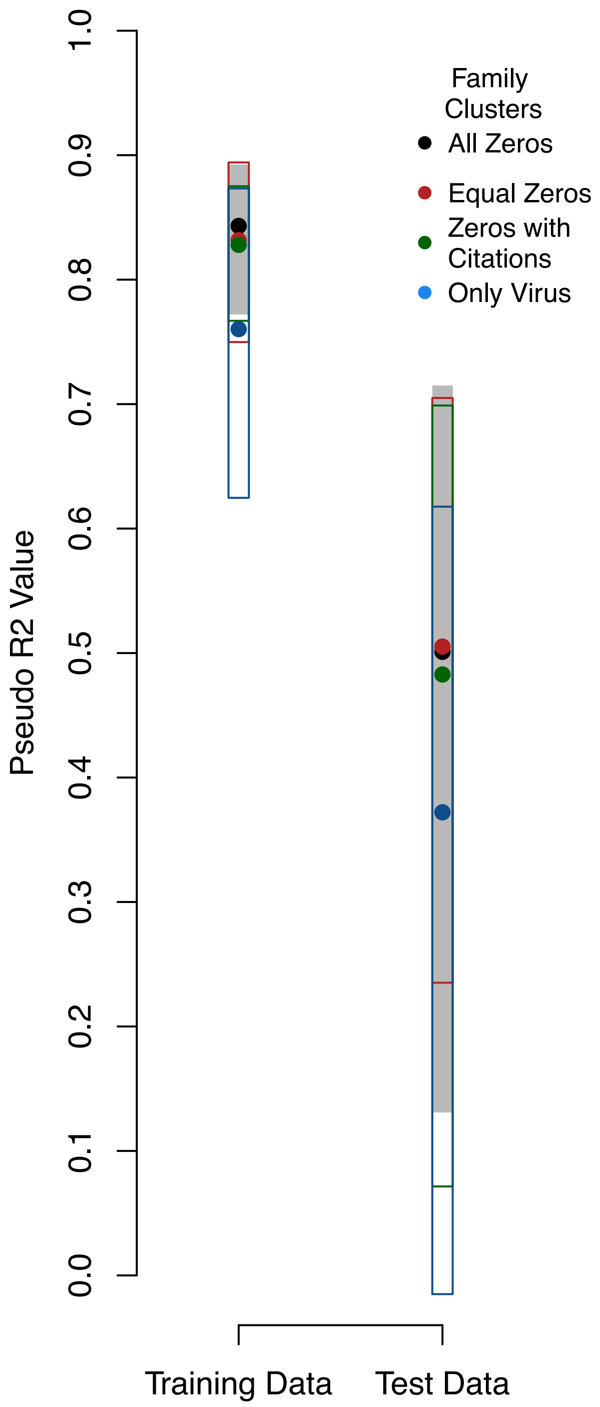

Figure B6. Measures of model performance from the BRT models built using the methods described above in [1-4]. Points represent average relative importance across 200 runs, while shaded or outlined bars represent the range within which 95% of values fall.

B.3 Effect of Phylogenetic Grouping on Boosted Regression Tree Models

Unlike standard phylogenetic regressions, BRTs cannot explicitly control for shared ancestry among species, which is known to bias other statistical methods (Felsenstein 1985, Harvey and Pagel 1991, Revell 2010). Although BRTs are a distribution-free method (Elith et al. 2008) and this has been used as a justification for their application to species level data (*i.e.*, Han *et al.* 2015, 2016), we explored how inclusion of phylogenetic information might impact conclusions drawn from BRT models, as well as their predictive accuracy. To do this, we ran several variations of our BRT models, incorporating phylogeny at three different levels, as outlined in the main text.

[5] Full Model & Phylogenetic Groupings: We split our full dataset, containing all species (n=747), into training (80% of species) and test (20% of species) datasets and built a BRT model. We included all ecological predictors and number of viral families as the response. We repeated this procedure separately for each of the three phylogenetic groupings outlined in the main text: family was included as a predictor in the first BRT model, the 56MYA phylogenetic clusters were included in the second BRT model, and the 41MYA phylogenetic clusters were included in the third BRT model. Unlike our procedure for examining the importance of zeros in BRT models ([2] above), we ran each model only once. For each model, we recorded measures of variable relative importance and model performance (*i.e.*, training and test pseudo R^2).

[6] Phylogeny and Model Accuracy: To explore if phylogenetic grouping biased model predictive performance we built BRT models on a subset of our phylogenetic groupings, and validated models using the withheld phylogenetic clusters. That is, instead of partitioning our whole dataset (n=747 species) into 80% training and 20% test datasets, we randomly selected a

subset of our phylogenetic clusters to act as the training dataset (still representing ~80% of bat species in the full dataset) and used the remaining phylogenetic clusters as a test dataset (still representing ~20% of bat species in the full dataset). We performed this random sampling procedure for each of the three phylogenetic groupings outlined in the main text: we took a random sampling of bat families, the 41MYA phylogenetic clusters, and the 56MYA phylogenetic clusters to build training datasets that contained ~80% of the full dataset. We then used this random drawing of clusters to build BRT models containing all ecological predictors and number of viral families as the response. However, unlike in [5] above, given that we were using our phylogenetic groupings to partition the dataset, we did not include them as predictors in the respective BRT models. Additionally, we ran each model only once (as above) since there were a limited number of cluster combinations in some phylogenetic groupings (*i.e.*, 56MYA) where the data could be split into 80% training and 20% test datasets. For each model, we recorded measures of variable relative importance and model performance.

Results

Similar to the results of [2] above (Fig. B3), including different phylogenetic groupings did not substantially alter the orderings of ecological predictors (Table B1). In all cases, the top 10 predictors, contributing to ~90% of the summed relative importance in the final models, remained the same (Fig. B3 & Table B1). There were slight shifts in the relative importance of species' relationships depending on the phylogenetic grouping included. In models run using a subset of zeros (see [2] above; Fig. B3) and models run using all zeros (see [5] above; Table B1) 41MYA clusters tended to have a greater relative importance than bat families, and bat families tended to have a greater relative importance than the 56MYA clusters. This likely has to do with the number of species contained within each cluster. The 41MYA clusters split species up into

smaller groupings (n= 42 clusters), increasing the likelihood that particular clusters contained more viral reservoirs than others. Conversely, the 56MYA clusters (n=7 clusters) contain a large number of species, making it less likely that cluster identity predicted viral diversity, as all groups may have some viral carriers given the number of species in each cluster. Family based groupings (n=20 clusters) fall in between the 41MYA clusters and 56MYA clusters in the number of groups into which it partitions the data. Given that family is the middle ground between our two phylogenetic clusterings, in main text models we include it as a predictor. Regardless, measures of model predictive performance were similar irrespective of which phylogenetic grouping was included as an ecological predictor in BRT models (Fig. B4 and Table B1).

When BRT models were built on a subset of phylogenetic clusters and validated on withheld clusters, there were small changes to the relative importance of certain ecological traits (Table B2). This was due to the fact that the phylogenetic clusters were not included in the model as ecological predictors. However, measures of model performance were similar to models built using a random selection of 80% of all species and including the phylogenetic clusters as variables (*e.g.* see [2] and [5] above; Table B1 & B2, Fig B4).

Conclusions

Although information about species relationships is consistently important in BRT models (*i.e.*, 41 MYA, bat families, and 56 MYA clusters were all within the top 10 ecological predictors of all BRT models), this information does not impact model predictive performance. In models discussed in the main text, we include family as our phylogenetic predictor given that it emphasizes similar ecological traits to the 41 MYA and 56 MYA clusters and that results may be more intuitive to interpret.

Table B1. Summarized results from boosted regression tree models containing different phylogenetic groupings. In all models, total viral family diversity was used as the response. Results are based on single BRT models, rather than 200 iterations. Optimized values for shrinkage, interaction depth, and number of trees are included along with measures of model performance. Phylogenetic groupings are highlighted in yellow.

	All Virus + Family		All Virus + 42MYA		All Virus + 56MYA	
**Interaction Depth**	4		4		3	
**Shrinkage**	0.0001		0.001		0.001	
**Total Trees**	60000		10000		70000	
**Best iteration**	57999		3951		70000	
**Pseudo R^2 (train)**	0.83		0.76		0.83	
**Pseudo R^2 (test)**	0.44		0.59		0.59	
	**Predictors**	**Rel.imp**	**Predictors**	**Rel.imp**	**Predictors**	**Rel.imp**
	sin_Citations	47.38	sin_Citations	41.16	sin_Citations	37.00
	Lifespan	9.38	PhyloClust41	18.33	Lifespan	13.01
	Median_X_Long	8.85	Lifespan	9.74	Median_X_Lon g	9.84
	Family	8.02	Median_X_Long	8.01	log_Median	8.75
	log_Median	6.23	sqrt_Rarea	5.75	sqrt_Rarea	7.33
	sqrt_Rarea	5.29	Median_Y_Lat	4.23	RWL	4.45
	Median_Y_Lat	3.42	log_Median	3.30	PhyloClust56	4.08
	RWL	3.03	RWL	2.88	log_AR	4.00
	log_AR	2.48	log_AR	1.52	Median_Y_Lat	3.70
	Residuals	1.90	Residuals	1.51	log_Forearm	2.32
	log_Forearm	1.66	Torpor	0.99	Torpor	1.58
	sin_No_Mixed	0.54	log_Forearm	0.92	Residuals	1.52
	Pulse	0.54	Trend	0.79	Trend	0.85
	Torpor	0.47	Pulse	0.40	Roost	0.55
	Trend	0.44	sin_No_Mixed	0.16	Pulse	0.39
	Roost	0.22	Roost	0.13	Migration	0.30
	Migration	0.10	Diet_2	0.07	sin_No_Mixed	0.26
	Status	0.02	Migration	0.07	Diet_2	0.04
	Diet_2	0.01	Status	0.04	Status	0.01

Table B2. Summarized results from boosted regression tree models built and tested on different phylogenetic groupings. In all models, total viral family diversity was used as the response. Results are based on single BRT models, rather than 200 iterations. Optimized values for shrinkage, interaction depth, and number of trees are included along with measures of model performance.

	All Virus; Family Groups		All Virus; 41MYA Clusters		All Virus; 56MYA Clusters	
**Shrinkage**	0.001		0.001		0.0001	
**Interaction Depth**	2		1		5	
**Total Trees**	15000		20000		70000	
**Best iteration**	14161		19955		54005	
**Pseudo R^2 (train)**	0.85		0.79		0.81	
**Pseudo R^2 (test)**	0.5		0.49		0.5	
	**Predictors**	**Rel.imp**	**Predictors**	**Rel.imp**	**Predictors**	**Rel.imp**
	sin_Citations	40.59	sin_Citations	46.18	sin_Citations	49.67
	Lifespan	14.09	Lifespan	18.69	Median_X_Long	14.39
	Median_X_Long	11.39	RWL	6.38	Lifespan	5.67
	log_Median	7.79	log_Median	6.37	log_AR	5.04
	sqrt_Rarea	5.84	Residuals	4.33	RWL	4.90
	RWL	5.56	Torpor	3.78	sqrt_Rarea	4.11
	Residuals	3.65	sqrt_Rarea	3.60	Median_Y_Lat	3.66
	log_Forearm	2.80	Median_X_Long	3.10	Residuals	3.08
	log_AR	2.27	log_AR	2.02	log_Forearm	2.69
	Median_Y_Lat	1.95	Median_Y_Lat	1.83	log_Median	2.06
	Torpor	1.49	log_Forearm	1.36	Trend	1.13
	Pulse	0.75	Pulse	1.23	Torpor	1.03
	Roost	0.63	sin_No_Mixed	0.65	Roost	0.88
	Trend	0.54	Status	0.21	sin_No_Mixed	0.82
	sin_No_Mixed	0.34	Migration	0.21	Pulse	0.51
	Migration	0.21	Trend	0.05	Migration	0.30
	Status	0.08	Roost	0.02	Status	0.03
	Diet_2	0.02	Diet_2	0.00	Diet_2	0.02

B.4: Effect of Additional Viral Information on Boosted Regression Tree Models

While previous studies have involved extensive literature searches (*e.g.*, Turmelle & Olival 2009; Luis *et al.* 2013) or collected their own viral data in the field (*e.g.* Maganga *et al.* 2014), similar to Webber *et al.* (2017) we gathered our data from an online repository of bat viral sequence data, DBatVir (Chen et al. 2014). We chose to leverage this dataset for two reasons. First, it is continually updated, thus our analysis can serve as a baseline against which future work could be compared as more information becomes available. Second, it is freely accessible, avoiding some of the replication bias due to variable access in literature searches (Dallas et al. 2018). However, a limitation of our choice to use DBatVir is that it contains only viral sequence data isolated through metagenomic approaches (Chen et al. 2014). As a result, it may not contain information on viruses detected in bats via other means.

To examine the potential effects of missing viral data on our models, we returned to an existing bat-viral dataset published in 2013 by Luis *et al.* This dataset contains many older references for bats and their viruses, obtained through an exhaustive literature search. Our choice of this dataset as reference was partially motivated by the fact that our models (built on DBatVir data) predicted three bat species (*i.e.*, *Barbastella barbastellus, Phyllostomus hastatus* and *Myotis grisescens*) currently "unknown" viral carriers according to DBatVir, that were listed as known carriers in Luis et al. (2013). To examine the effects of this additional information from non-DBatVir sources on model inferences we performed the following analyses:

[7] Removal of all Luis Species: There were a total of 44 species for which the Luis *et al.* (2013) dataset contained viral information, but for which we had no sequence data from DBatVir. For the first set of analyses we removed these species completely from our dataset. We then used the remaining 703 bat species to build 200 BRT models for total viral family diversity

using all ecological predictors as outlined in the main text. We used these models to examine the relative importance of traits for prediction and the predictive accuracy of models. We also built 200 BRT models for total viral family diversity using the residual approach (controlling for the effects of citations) outlined in the main text. We compared the resulting predictions within the 95th and 99th percentiles from these models to those predictions generated from our complete models discussed in the main text.

[8] Addition of Luis Data: Instead of removing 44 bat species in the Luis *et al.* (2013) dataset, we included the information from Luis *et al.* (2013) (*i.e.*, updating some of the zeros in our dataset). We used the resulting 747 species (251 viral carriers, 496 non-viral carriers) to build 200 BRT models for total viral family diversity using all ecological predictors as outlined in the main text. We used these models to examine the relative importance of traits for prediction and the predictive accuracy of models. We also built 200 BRT models for total viral family diversity using the residual approach outlined in the main text. We compared the resulting predictions within the 95th and 99th percentiles from these models to those predictions generated from our complete models discussed in the main text.

Results

Including viral information from the Luis *et al.* (2013) dataset [8] or removing these species completely [7] did not affect the relative importance of bat ecological traits or the predictive accuracy of models (Figs. B7 and B8). These models emphasized the same traits as being important as the main text models (Fig. B7). Given that we either removed bat species [7] or updated viral information for some bat species [8] there were changes to the bats predicted to carry viruses by our models. Eleven of the species in our 90th percentile of model predictions were in the Luis *et al.* (2013) dataset. However, all of the species in the 95th percentile of model

predictions from [7] and [8] were in the 90th percentile of model predictions for the main text models.

Conclusions

The fact that our models identify species included in the Luis *et al.* (2013) dataset as potential viral carriers suggests our models are predicting well. While inclusion of more viral information will result in shifts in species predicted as carriers, the fact that all species in the 95th percentile of model predictions built using the Luis *et al.* (2013) data are in the 90th percentile predictions from our main text model (that has Luis *et al.* (2013) species listed as zeros) would indicate that these species are indeed important potential reservoirs. As such, in the main text we discuss model predictions using our model built exclusively with the DBatVir data.

Table B3. Bat species predicted to be undetected viral carriers in the 99th percentile by the main text BRT models and BRT models described in [7] and [8] above. Species listed as non-carriers according to DBatVir, for which there was viral data in Luis *et al.* (2013) are bolded.

Percentile	Predicted Reservoir Species		
	Main Text Model	Removal of Luis Species [7]	Inclusion of Luis Species [8]
99th	*Asellia tridens*	*Asellia tridens*	*Asellia tridens*
	Barbastella barbastellus	*Coelops frithii*	*Coelops frithii*
	Coelops frithii	*Myotis sodalis*	*Myotis keaysi*
	Myotis grisescens	*Pteropus rodricensis*	*Myotis sodalis*
	Phyllostomus hastatus	*Rhinopoma microphyllum*	*Pteropus rodricensis*
	Pteropus rodricensis	*Chalinolobus gouldii*	

Table B4. Bat species predicted to be undetected viral carriers in the 95th percentile by the main text BRT models and BRT models described in [7] and [8] above. Species listed as non-carriers according to DBatVir, for which there was viral data in Luis *et al.* (2013) are bolded. All predicted reservoirs species in [7] and [8] which were also in the 90th percentile predictions in the main text model are marked with an asterisk (*).

Percentile	Predicted Reservoir Species		
	Main Text Model	Removal of Luis Species [7]	Inclusion of Luis Species [8]
95th	*Chalinolobus gouldii*	*Chalinolobus morio*	*Rhinopoma microphyllum*
	Chalinolobus morio	*Haplonycteris fischeri*	*Chalinolobus gouldii*
	Eumops perotis	*Hipposideros cervinus*	*Chalinolobus morio*
	Haplonycteris fischeri	*Hipposideros fulvus*	*Haplonycteris fischeri*
	Hipposideros cervinus	*Hipposideros galeritus**	*Hipposideros fulvus*
	Hipposideros fulvus	*Hipposideros lylei*	*Hipposideros galeritus**
	Hipposideros lylei	*Leptonycteris yerbabuenae*	*Hipposideros lylei*
	Hipposideros terasensis	*Macroderma gigas**	*Leptonycteris yerbabuenae*
	Macroglossus minimus	*Myotis adversus*	*Macroderma gigas**
	Mormoops megalophylla	*Myotis brandtii*	*Myotis adversus*
	Myotis adversus	*Myotis keaysi*	*Myotis brandtii*
	Myotis brandtii	*Neoromicia tenuipinnis*	*Noctilio leporinus*
	Myotis keaysi	*Nyctimene albiventer**	*Nyctimene albiventer**
	Myotis sodalis	*Nyctimene robinsoni**	*Nyctimene robinsoni**
	Nyctophilus gouldi	*Nyctophilus gouldi*	*Nyctophilus gouldi*
	Platyrrhinus lineatus	*Rhinolophus formosae**	*Rhinolophus capensis*
	Rhinolophus paradoxolophus	*Rhinolophus paradoxolophus*	*Rhinolophus formosae**
	Rhinolophus yunnanensis	*Rhinolophus yunnanensis*	*Rhinolophus paradoxolophus*
	Rhinopoma microphyllum	*Tadarida aegyptiaca*	*Rhinolophus yunnanensis*
	Syconycteris australis		*Tadarida aegyptiaca*
	Tadarida aegyptiaca		

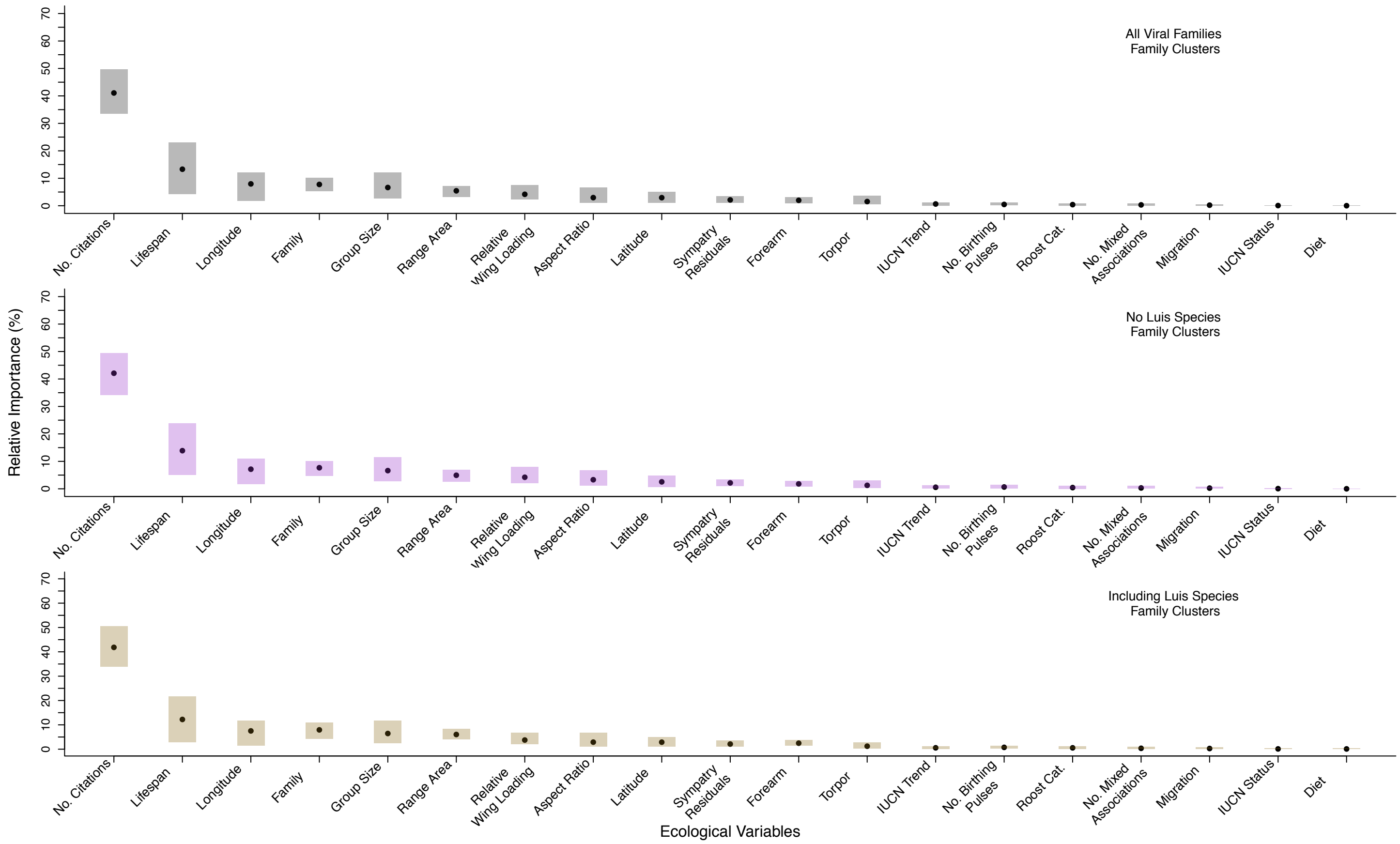

Figure B7. Relative importance of ecological traits from 200 BRT model runs. Points represent average relative importance across runs, while shaded bars represent the range within which 95% of values fall. ***Top Panel:*** Relative importance measures for predicting total viral family diversity with all ecological predictors. ***Middle Panel:*** Relative importance measures for predicting total viral family diversity excluding species in the Luis *et al.* (2013) dataset for which they have viral information, but we had no molecular sequence data (*i.e.*, 44 of the zeros in our dataset). ***Bottom Panel:*** Relative importance measures for predicting total viral family diversity including viral information for species in the Luis *et al.* (2013) dataset for which we had no molecular sequence data (*i.e.*, 'updating' those zeros).

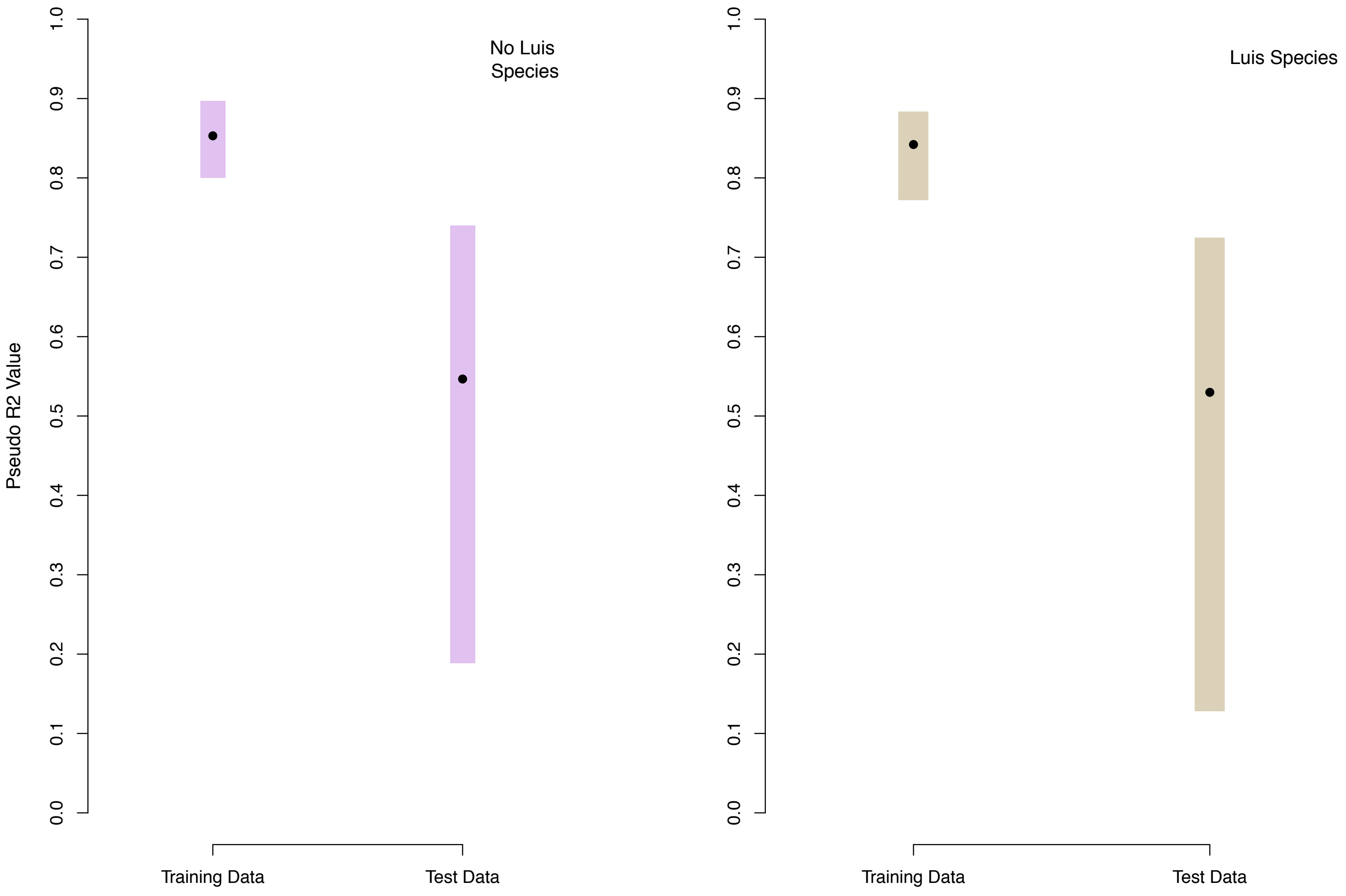

Figure B8. Measures of model performance from the 200 BRT model runs built using the full dataset. Points represent average pseudo R^2 across all runs, while shaded bars represent the range within which 95% of values fall within. ***Left Panel:*** Model performance for predicting total viral family diversity with all ecological predictors, but excluding species in the Luis *et al.* (2013) dataset for which they have viral information, but we have no molecular sequence data (*i.e.*, 44 of the zeros in our dataset). ***Right Panel:*** Model performance for predicting total viral family diversity with all ecological predictors, but including viral information for species in the Luis *et al.* (2013) dataset for which we had no molecular sequence data (i.e., 'updating' those zeros).

Works Cited

Chen, L., B. Liu, J. Yang, and Q. Jin. 2014. DBatVir: the database of bat-associated viruses. Database The Journal of Biological Databases and Curation 2014:bau021.

Dallas, T., A.-L. Gehman, and M. J. Farrell. 2018. Variable Bibliographic Database Access Could Limit Reproducibility. BioScience 68:552–553.

Elith, J., J. R. Leathwick, and T. J. Hastie. 2008. A working guide to boosted regression trees. Journal of Animal Ecology 77:802–813.

Felsenstein, J. 1985. Phylogenies and the comparative method. The American Naturalist 125:1–15.

Han, B. A., J. P. Schmidt, L. W. Alexander, S. E. Bowden, D. T. S. Hayman, and J. M. Drake. 2016. Undiscovered bat hosts of filoviruses. PLoS Neglected Tropical Diseases 10:1–10.

Han, B. A., J. P. Schmidt, S. E. Bowden, and J. M. Drake. 2015. Rodent reservoirs of future zoonotic diseases. Proceedings of the National Academy of Sciences 112:7039–44.

Harvey, P. H., and M. Pagel. 1991. The Comparative Method in Evolutionary Biology. Oxford University Press, Oxford.

Kuhn, M., J. Wing, S. Weston, A. Williams, C. Keefer, A. Engelhardt, T. Cooper, Z. Mayer, B. Kenkel, the R. C. Team, M. Benesty, R. Lescarbeau, A. Ziem, L. Scrucca, Y. Tang, C. Candan, and T. Hunt. 2018. "caret": Classification and Regression Training.

Lindenfors, P., C. L. Nunn, K. E. Jones, A. A. Cunningham, W. Sechrest, and J. L. Gittleman. 2007. Parasite species richness in carnivores: effects of host body mass, latitude, geographical range and population density. Global Ecology and Biogeography 16:496–509.

Luis, A. D., D. T. S. Hayman, T. J. O'Shea, P. M. Cryan, A. T. Gilbert, J. R. C. Pulliam, J. N. Mills, M. E. Timonin, C. K. R. Willis, A. A. Cunningham, A. R. Fooks, C. E. Rupprecht, J.

L. N. Wood, and C. T. Webb. 2013. A comparison of bats and rodents as reservoirs of zoonotic viruses: Are bats special? Proceedings of the Royal Society B: Biological Sciences 280:20122753.

Maganga, G. D., M. Bourgarel, P. Vallo, T. D. Dallo, C. Ngoagouni, J. F. Drexler, C. Drosten, E. R. Nakouné, E. M. Leroy, and S. Morand. 2014. Bat distribution size or shape as determinant of viral richness in african bats. PLoS ONE 9:e100172.

Nunn, C. L., S. Altizer, K. E. Jones, and W. Sechrest. 2003. Comparative tests of parasite species richness in primates. The American Naturalist 162:597–614.

Olival, K. J., P. R. Hosseini, C. Zambrana-Torrelio, N. Ross, T. L. Bogich, and P. Daszak. 2017. Host and viral traits predict zoonotic spillover from mammals. Nature 546:646–650.

R Core Development Team. 2014. R: A language and environment for statistical computing. R Foundation for Statistical Computing, Vienna, Austria.

Revell, L. J. 2010. Phylogenetic signal and linear regression on species data. Methods in Ecology and Evolution 1:319–329.

Ridgeway, G. 2017. “gbm”: Generalized Boosted Regression Models.

Shi, J. J., and D. L. Rabosky. 2015. Speciation dynamics during the global radiation of extant bats. Evolution 69:1528–1545.

Turmelle, A. S., and K. J. Olival. 2009. Correlates of viral richness in bats (Order Chiroptera). EcoHealth 6:522–539.

Webber, Q. M. R., Q. E. Fletcher, and C. K. R. Willis. 2017. Viral richness is positively related to group size, but not mating system, in bats. EcoHealth 14:652–661.
